# Comparative analysis of swine leukocyte antigen gene diversity in Göttingen Minipigs

**DOI:** 10.3389/fimmu.2024.1360022

**Published:** 2024-02-26

**Authors:** Sabine E. Hammer, Tereza Duckova, Monica Gociman, Sandra Groiss, Clara P. S. Pernold, Karolin Hacker, Lena Kasper, Julia Sprung, Maria Stadler, Andres Eskjær Jensen, Armin Saalmüller, Nadine Wenzel, Constanca Figueiredo

**Affiliations:** ^1^ Department of Pathobiology, Immunology, University of Veterinary Medicine Vienna, Vienna, Austria; ^2^ Institute of Transfusion Medicine and Transplant Engineering, Hannover Medical School, Hannover, Germany; ^3^ Merck Healthcare KGaA, Darmstadt, Germany; ^4^ Ellegaard Göttingen Minipigs A/S, Dalmose, Denmark

**Keywords:** *Sus scrofa*, swine leukocyte antigen (SLA), polymorphism, animal model, biomedical research and development, transplantation, xenograft

## Abstract

Worldwide, pigs represent economically important farm animals, also representing a preferred preclinical large animal model for biomedical studies. The need for swine leukocyte antigen (SLA) typing is increasing with the expanded use of pigs in translational research, infection studies, and for veterinary vaccine design. Göttingen Minipigs (GMP) attract increasing attention as valuable model for pharmacological studies and transplantation research. This study represents a first-time assessment of the SLA gene diversity in Göttingen Minipigs in combination with a comparative metadata analysis with commercial pig lines. As Göttingen Minipigs could harbor private as well as potential novel SLA allele combinations, future research projects would benefit from the characterization of their SLA background. In 209 Göttingen Minipigs, SLA class I (*SLA-1*, *SLA-2*, *SLA-3*) and class II (*DRB1*, *DQB1*, *DQA*) genes were characterized by PCR-based low-resolution (Lr) haplotyping. Criteria and nomenclature used for SLA haplotyping were proposed by the ISAG/IUIS-VIC SLA Nomenclature Committee. Haplotypes were assigned based on the comparison with already known breed or farm-specific allele group combinations. In total, 14 SLA class I and five SLA class II haplotypes were identified in the studied cohort, to manifest in 26 SLA class I but only seven SLA class II genotypes. The most common SLA class I haplotypes Lr-24.0 (SLA-1*15XX or Blank-SLA-3*04:04-SLA-2*06:01~02) and Lr-GMP-3.0 (SLA-1*16:02-SLA-3*03:04-SLA-2*17:01) occurred at frequencies of 23.44 and 18.66%, respectively. For SLA class II, the most prevalent haplotypes Lr-0.21 (DRB1*01XX-DQB1*05XX-DQA*04XX) and Lr-0.03 (DRB1*03:02-DQB1*03:01-DQA*01XX) occurred at frequencies of 38.28 and 30.38%. The comparative metadata analysis revealed that Göttingen Minipigs only share six SLA class I and two SLA class II haplotypes with commercial pig lines. More importantly, despite the limited number of SLA class I haplotypes, the high genotype diversity being observed necessitates pre-experimental SLA background assessment of Göttingen Minipigs in regenerative medicine, allo-transplantation, and xenograft research.

## 1 Introduction

Although we can attribute to the mouse a very huge list of accomplishments related to science research and crucial discoveries that have made a huge impact in human’s lives, mice have limitations that scientists have been aware of and the physiological differences that set apart mice and humans still represent a big gap that cannot be easily overcome. Animal models are essential to bridge the gap between preclinical and clinical research and when searching for an animal model criteria like affordability and accessibility are crucial aspects to have in mind (1–3). Although mice fulfill these requirements they also differ considerably from humans in aspects like size, lifespan, metabolism, physiology and in their immunological background. Setting aside nonhuman primates that are linked to complex ethical issues and high costs, focus has turned upon other animal models among which dogs, cats, cattle, and pigs are the most frequently used for research purposes (4, 5). Over the past two decades there has been a significant increase in the interest of using pigs in translational research which has led to an enhanced understanding of human’s diseases and has also contributed to improvements in human health (6, 7). Swine are also used as general surgical models and for human diseases, for development of biopharmaceuticals and more recently, implantable medical devices (8). Recent discovered technologies such as CRISPR/Cas9 (clustered regularly interspaced short palindromic repeats/CRISPR-associated protein 9) made possible to genetically engineer pigs in human diseases such as Cystic fibrosis, Duchenne muscle dystrophy, cardiovascular diseases, and colorectal cancer (6, 7). Similarities between swine and humans regarding immune system and the physiology of the lymphatic system as well as the skin and the mucosal tissue supports the use of swine as animal model for immunological studies, including for T-cell research. Over the past decades many scientists have dedicated their research to characterizing the different T-cell subsets of the pigs and there have been constant updates meant to decipher the specificity of T cells (9). All the in-depth analysis and research performed in the past years provides the swine with an excellent ability to drive biomedical research even further including in vaccine development and not only (4, 5).

Minipigs possess many advantages that surpass many other animal models used in research (8, 10). From small size making them easy to handle, reduced requirements regarding food, space, pharmacologic products, convenient body size for use in surgical procedures (11). Minipigs are purpose bred for research and there are many breeds available world-wide. Göttingen Minipigs are a result of a crossing of three different breeds at the University of Göttingen. The breeding process dates from the 1960 when they were imported from the Hormel Institute, Austin, USA and crossbred with Vietnamese potbelly pigs in 1965 and German Landrace, their breeding being completed in 1969 (12). Göttingen Minipigs have a well-defined genetic background and physiological parameters, they share similarities with humans in terms of genetics, genomics, and biochemistry so they have become the main choice in allo- and xenotransplantation studies (6, 13–16). Additionally, in toxicological testing of therapeutic recombinant antibodies Minipigs that are tolerant to most human recombinant antibodies have been generated (17).

Understanding the regulation of the immune system and identifying those genes that are involved in these processes is crucial in biomedical research. The highly polymorphic porcine SLA complex is associated with different levels of immunologic responses to infectious diseases, vaccines, and transplantation. SLA-typed pigs are important genetic resources for biomedical research and using pigs with the same genetic background can facilitate genetic mapping. Confirmed allelic variants of SLA genes have been found to bind different classes of peptides. Therefore, so-called SLA typing is necessary to understand the underlying molecular mechanisms of SLA antigen binding and presentation. The determination of expressed SLA alleles represents an essential initial step in identifying virus-derived T-cell epitopes that play a role in generating protective T-cell responses against infections in pigs (18; reviewed in 19). Information on occurring SLA alleles could also influence the future vaccine design because if a limited number of SLA class I and class II genes are dominant, there is also the possibility they would present peptides that were conserved across viral strains, and this could lead to potential peptides used in targeting of T-lymphocyte responses which will benefit vaccine development (20, 21). All the progress made in pig research will ensure the continuous development of various pig strains meant to ensure similar advances to what has been achieved in mice (reviewed in 5, 19).

The Institute of Transfusion Medicine and Transplant Engineering from the Hannover Medical School (MHH, Germany) has a long collaborating history with the Institute of Immunology at the University of Veterinary Medicine Vienna on SLA typing mismatch donor pairs of Göttingen Minipigs for allo-transplantation research. Previous studies provided evidence for potential novel and so-called private SLA class I and SLA class II haplotypes in the studied cohorts (22–24). A total number of 209 Göttingen Minipigs have been genotyped for their SLA class I and class II haplotypes by running low-resolution PCR screening assays to make use of these animals in ongoing research projects. This is the first comparative metadata analysis between Göttingen Minipigs and farmed pig lines being performed since the establishment of PCR-based typing approaches. By applying a total of 94 allele-group specific primer pairs it was possible to characterize the SLA class I and SLA class II background of 209 Göttingen Minipigs.

## 2 Materials and methods

### 2.1 Animals and sample collection

Throughout the years 2016 until 2023, we analyzed and compiled the SLA diversity data of 209 Göttingen Minipigs (GMP) from the company Ellegaard Göttingen Minipigs A/S (Dalmose, Denmark). Based on their chronological origin, the animals were divided in six major collaborative projects (‘cohorts’) and listed in **Supplementary Table 1** along with the internal identification (ID) number for each pig. Cohort 2016, 2017, and 2018 consisted of 17, 16, and 30 pigs, whereas cohort 2019, 2021, and 2023 were composed of 67, 34, and 45 Göttingen Minipigs, respectively. From most of the studied cohorts, whole blood samples were received from Ellegaard Göttingen Minipigs A/S and subjected to DNA extraction (22–24). For some collaborative projects, isolated peripheral blood mononuclear cells (PBMC) or ready-to-use genomic DNA (gDNA) was shipped to our lab for SLA typing (22–24).

The corresponding animal studies at our partner institutions were approved as follows: in Denmark by the Danish authorities (license 2019-15-0201-01622), in Austria by the institutional ethics committee, the Advisory Committee for Animal Experiments (§ 12 Animal Experiments Act - TVG), and the Austrian Federal Ministry of Education, of Science and Research (reference BMBWF-68.205/0198-V/3b/2019) (10). Animal experiments being conducted at the Hannover Medical School (Germany) were licensed under the following reference numbers: 33.8-42502-04-16/2333 (Ellegaard Minipigs, LAVES, Deutschland), and 2023-15-0201-01352 (Danish authorities) (25).

### 2.2 DNA extraction and SLA typing by PCR-SSP

In total, 209 Göttingen Minipigs were genotyped for their swine leukocyte antigen (SLA) class I and II haplotypes by running low resolution PCR screening assays (PCR-SSP) on PBMC-derived genomic DNA. Animals originate from crossbreeding regimes of the lines Minnesota minipig, Vietnamese pot-bellied pig and German Landrace (12). Total genomic DNA (gDNA) was isolated from whole blood or peripheral blood mononuclear cells (PBMCs) using commercial kits following the manufacturers’ instructions (DNeasy Blood & Tissue Kit, Qiagen GmbH, Hilden, Germany; E.Z.N.A.^®^ Tissue DNA Kit, Omega Bio-tek, Inc., Norcross, GA, USA; NucleoSpin^®^ Blood XL, Macherey-Nagel, Düren, Germany). Typing PCR reactions contained 1x HotStarTaq™ Plus master mix (Qiagen), 1x CoralLoad loading buffer (Qiagen), 0.2 pmol/µL of α-actin positive control primers, 0.2 pmol/µL of allele-specific primers (Eurofins Genomics, Ebersberg, Germany) and 20 ng of DNA, in a total volume of 10 µL. Typing of each pig included a negative control without DNA to check for reagent contamination, and was set up and electrophoresed in a standard 96-well format (26–29). The thermal-cycling conditions on the T Gradient thermal cycler (Biometra, Göttingen, Germany) consisted of an initial incubation of 95°C for 5 min, followed by 30 cycles of 95°C for 30 s, 65°C for 30 s and 72°C for 30 s. PCR products were electrophoresed in 2.5% DNA grade agarose gels (Biozym Biotech Trading GmbH, Vienna, Austria) in 1x TAE buffer at 150 V for 5 min using the Micro SSP Gel System (One Lambda, Canoga Park, CA, USA) and visualized after staining with GelStar™ (Lonza, Rockland, ME, USA). Interpretation of the results was based on the presence of allele-specific PCR products of the expected size in each lane. The criteria and nomenclature used for SLA-I and SLA-II haplotyping were based on those proposed by the SLA Nomenclature Committee (19, 30, 31). Interpretation of the results was deduced from the presence of allele-specific PCR products of the expected size in each lane. Low-resolution SLA class I and class II haplotypes were assigned based on the comparison with previously published haplotypes (27–29, 32–35) and unpublished breed or farm-specific haplotypes (C.-S. Ho et al., S.E. Hammer et al., unpublished data).

### 2.3 Calculation of haplotype, genotype, and allele-group frequencies

Among the 209 studied Göttingen Minipigs, different haplotypes and genotypes combinations were assigned and based on these results, we estimated the frequencies of SLA class I and class II allele groups and haplotypes together with SLA class I/II genotype combinations according to their occurrence among the studied cohorts.

Calculation of haplotype and allele-group frequencies is based on the percentage of SLA class I and class II low resolution haplotypes and allele-groups among two times the number of studied animals. The corresponding formula is as follows (x = number of occurring haplotypes, n = total number of studies animals):


$$\text{Freq}\left(\%\right)=\left(\frac{\text{x}}{2\text{n}}\right)\times 100$$

Frequencies of SLA class I and class II single and combined genotypes is represented by the percentage of genotypes among the number of studied animals. The corresponding formula is as follows (y = number of occurring genotypes, n = total number of studies animals):


$$\text{Freq}\left(\%\right)=\left(\frac{y}{n}\right)\times 100$$

The different combinations between the assigned genotypes of SLA class I and class II are represented by the number of specific combinations occurring among all the studied animals.

## 3 Results

### 3.1 DNA concentration and quality of studied animals

After gDNA extraction or arrival of gDNA samples in our lab, their concentration and quality were assessed with the NanoDrop 2000c spectrophotometer (Thermo Fisher Scientific, Waltham, MA, USA). The results of gDNA concentration and quality measurements are summarized in **Supplementary Table 2**. The gDNA concentrations of 209 Göttingen Minipigs ranged from 12.20 to 508 ng/µl, with the average value of 117.98 ng/µl. The 260/280 ratios of the examined animals ranged from 1.68 to 2.29, with the average value of 1.96. The 260/230 ratios varied between 0.76 and 4.10 with a mean value of 1.96. We saw that the performance of PCR-SSP reactions was impaired in those samples with deviating 260/280 and/or 260/230 ratios because of unknown contaminants or low quality of reagents. Finally, for most of the studied pigs, the obtained quality measures reflected the outcome of the PCR-SSP assay in terms of resolution of the samples on the agarose gels.

### 3.2 SLA class I diversity in Göttingen Minipigs

**Figure 1:**
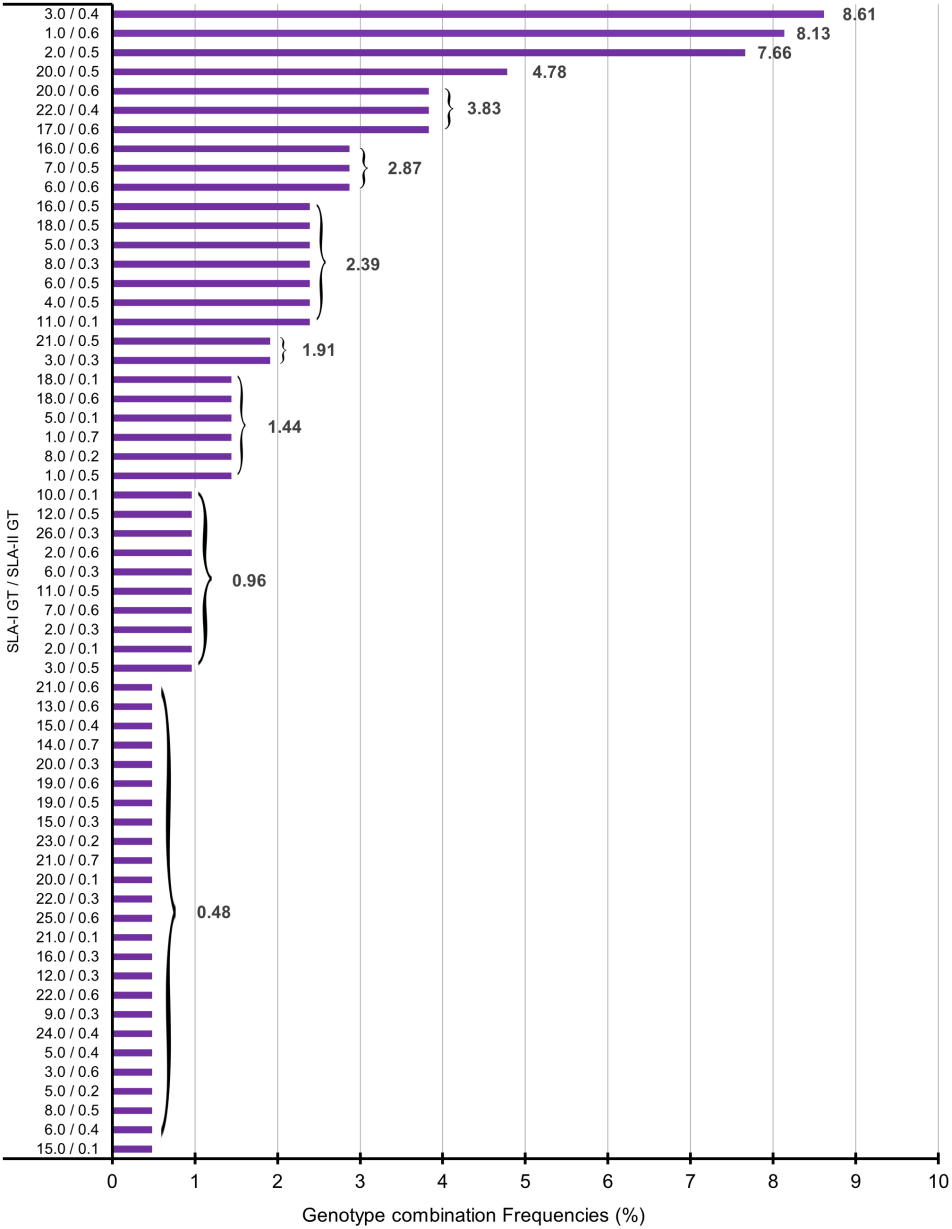
Graphical representation of combined genotypes in 209 Göttingen Minipigs. SLA, Swine Leucocyte Antigen; GT, Genotype.

**Figure 2:**
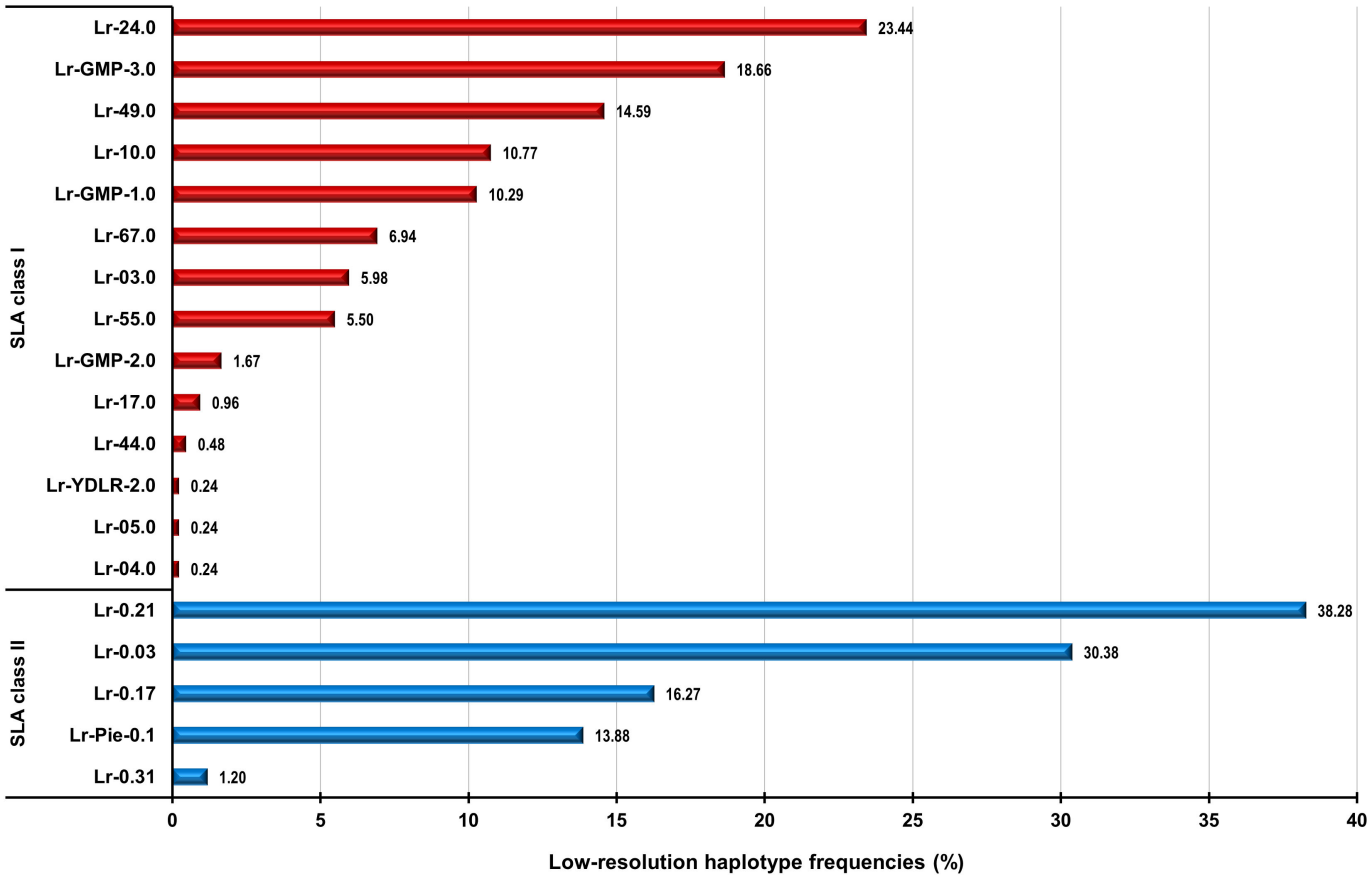
Graphical representation of frequencies of found SLA class I (indicated in red) and class II (indicated in blue) haplotypes in 209 Göttingen Minipigs. SLA, Swine Leucocyte Antigen; GMP, Göttingen Minipig; Lr, Low resolution Haplotype; YDLR, Yorkshire/Duroc/Landrace 3-way crossbreed; Pie, Pietrain.

The evaluation of each of the 209 digitized gel images revealed twenty-six different genotypes occurring in the six cohorts of Göttingen Miniature pigs (22–24). Assigned genotypes with corresponding allele groups and low-resolution haplotypes (Lr-Hp) for the SLA class I of the investigated animals are displayed in **Table 1**. Every investigated cohort had a minimum of three different genotypes. The cohort 2021 exhibited the maximum of thirteen different defined genotypes. Göttingen Minipig (GMP) genotype (GT) 3.0 was the most common SLA class I genotype occurring in 25 of 209 investigated Minipigs at a frequency of 11.96% (**Table 1**; **Supplementary Figure 1**). The SLA class I genotype GT-3.0 with its corresponding Lr-24.0 and Lr-49.0, Lr-24.0mod and Lr-49mod, was found in four out of six cohorts of this project, with the highest abundance in cohort 2019 with 12 out of 67 animals. Genotype GT-1.0 with the combination of haplotypes Lr-24.0 and GMP-1.0, Lr-24.0mod and GMP-1.0, represented the second most common genotype for SLA class I among the 209 minipigs and was found in 23 animals at a frequency of 11.00% (**Table 1**; **Supplementary Figure 1**). Twenty-two Minipigs were positive for the third most frequent SLA class I genotype GT-2.0 (frequency: 10.52%) with the combination of haplotypes Lr-10.0 and Lr-67.0mod, which was distributed among three out of six cohorts, cohort 2016, 2018, and 2019 (**Supplementary Table 6**; **Figure 1**). There were also genotypes that appeared only once, namely the genotype GT-9.0 (Lr-03.0mod and Lr-17.0), GT-13.0 (Lr-5.0 and Lr-GMP-3.0), GT-14.0 (Lr-YDLR-2.0 and Lr-49.0mod), GT-23.0 (Lr-49.0), GT-24.0 (Lr-17.0 and Lr-49.0), and GT-25.0 (Lr-04.0 and Lr-GMP-3.0). Other genotypes occurred in 2 to 20 animals according to **Table 1** and **Supplementary Figure 1**. Genotypes GT-9.0, GT-13.0, GT-14.0, GT-23.0, GT-24.0, and GT-25.0 occurred at the lowest frequency of 0.47%. The depicted allele groups gave rise to 14 class II haplotypes as depicted in **Supplementary Table 3**; **Figure 2**. The most frequent low-resolution haplotype in SLA class I was Lr-24.0 [SLA-1*Blank-SLA-3*04XX-SLA-2*06XX(06:01~02)] occurring at a frequency of 23.44%. Note: ‘Blank’ indicates alleles that cannot be identified with the primer sets being used in this study. With a frequency of 18.66%, Lr-GMP-3.0 [SLA-1*16:02-SLA-3*03XX(03:04)-SLA-2*17:01] was the second most abundant haplotype (**Supplementary Table 3**; **Figure 2**). Next, Lr-49.0 occurred with a frequency of 14.59%. The frequencies for Lr-10.0 and Lr-GMP-1.0 were 10.77% and 10.29%, respectively. Lower frequencies were found for Lr-67mod (6.94%), Lr-3.0 (5.98%), and Lr-55.0 (5.50%). Lr-GMP-2.0, Lr-17.0, and Lr-44mod occurred at frequencies of 1.67%, 0.96%, and 0.48%, respectively. Finally, Lr-YDLR-2.0, Lr-04.0 and Lr-05.0 were found with a frequency of 0.24% in all studied animals (**Supplementary Table 3**; **Figure 2**).

**Table 1:** SLA class I genotypes and haplotypes found in 209 Göttingen Minipigs.

SLA-1	SLA-3	SLA-2	Lr-Hp	GT	No’s	Frq(%)
Blank	04XX	06XX(06:01~02)	24.0	1.0	23	11.00
05XX/15XX	05XX/08XX	01XX	GMP-1.0			
05XX/15XX	04XX	06XX(06:01~02)	24.0mod			
05XX/15XX	05XX/08XX	01XX	GMP-1.0			
05XX	08XX	03XX	10.0	2.0	22	10.52
15XX	05XX	01XX	67.0mod			
Blank	04XX	06XX(06:01~02)	24.0	3.0	25	11.96
08XX	05XX	Blank	49.0			
05XX/15XX	04XX	06XX(06:01~02)	24.0mod			
08XX	05XX	01XX	49.0mod			
05XX/15XX	04XX	06XX(06:01~02)	24.0mod	4.0	5	2.39
05XX/15XX	08XX	01XX/03XX	GMP-2.0			
Null	03XX	03XX(03:01~05/03:08~09)	3.0	5.0	10	4.78
08XX	05XX	Blank	49.0			
15XX	03XX	03XX(03:01~05/03:08~09)	03.0mod			
08XX	05XX	06XX	49.0mod			
15XX	03XX	03XX(03:01~05/03:08~09)	03.0mod			
08XX	05XX	Blank	49.0			
Blank	04XX	06XX(06:01~02)	24.0	6.0	14	6.69
15XX	04XX	11:04	55.0			
05XX	08XX	03XX	10.0	7.0	8	3.82
Blank	04XX	06XX (06:01~02)	24.0			
05XX	08XX	03XX	10.0			
05XX/15XX	04XX	06XX (06:01~02)	24.0mod			
05XX	08XX	blank	10.0mod			
05XX/15XX	04XX	06XX(06:01~02)	24.0mod			
08XX	05XX	Blank	49.0	8.0	9	4.3
15XX	04XX	11:04	55.0			
Blank	04XX	11:04	55.0mod			
08XX	05XX	Blank	49.0			
11XX	03XX	03XX(03:01~05/03:08~09)	03.0mod	9.0	1	0.47
08XX	03XX	06XX	17.0			
Null	03XX	03XX(03:01~05/03:08~09)	3.0	10.0	2	0.95
Null	03XX	03XX(03:01~05/03:08~09)	3.0			
15XX	03XX	03XX(03:01~05/03:08~09)	03.0mod	11.0	7	3.34
Blank	04XX	06XX(06:01~02)	24.0			
Null	03XX	03XX(03:01~05/03:08~09)	3.0			
05XX/15XX	04XX	06XX(06:01~02)	24.0mod			
Null	03XX	03XX(03:01~05/03:08~09)	3.0	12.0	3	1.43
16:02	03XX(03:04)	03XX/17:01	GMP-3.0			
04XX	05XX	08XX	5.0	13.0	1	0.47
16:02	03XX(03:04)	03XX/17:01	GMP-3.0			
16:02(16:03)	04XX/04:04	06XX(06:04)	YDLR-2.0	14.0>	1	0.47
08XX	05XX	01XX	49.0mod			
05XX	08XX	03XX	10.0	15.0	3	1.43
08XX	05XX	blank	49.0			
05XX	08XX	03XX	10.0			
08XX	05XX	01XX	49.0mod			
05XX	08XX	03XX	10.0	16.0	12	5.74
16:02	03XX(03:04)	03XX/17:01	GMP-3.0			
05XX	08XX	Blank	10.0mod.			
16:02	03XX(03:04)	03XX/17:01	GMP-3.0			
Blank	04XX	06XX(06:01~02)	24.0	17.0	8	3.82
Blank	04XX	06XX(06:01~02)	24.0			
16:02	03XX(03:04)	03XX/17:01	GMP-3.0	18.0	11	5.26
16:02	03XX(03:04)	03XX/17:01	GMP-3.0			
16:02	03XX(03:04)	03XX/17:01	GMP-3.0	19.0	2	0.95
05XX/15XX	08XX	01XX/03XX	GMP-2.0			
16:02	03XX(03:04)	03XX/17:01	GMP-3.0	20.0	20	9.56
05XX/15XX	05XX/08XX	01XX	GMP-1.0			
16:02	03XX(03:04)	03XX/17:01	GMP-3.0	21.0	7	3.34
15XX	05XX	01XX	67.0mod			
16:02	03XX(03:04)	03XX/17:01	GMP-3.0	22.0	10	4.78
08XX	05XX	Blank	49.0			
16:02	03XX(03:04)	03XX/17:01	GMP-3.0			
08XX	05XX	01XX	49.0mod			
08XX	05XX	Blank	49.0	23.0	1	0.47
08XX	05XX	Blank	49.0			
08XX(08:04)	03XX(03:04)	06XX(06:03)	17.0	24.0	1	0.47>
08XX	05XX	blank	49.0			
04XX	04XX	04XX	4.0	25.0	1	0.47
16:02	03XX(03:04)	03XX/17:01	GMP-3.0			
08XX	03XX	06XX	17.0	26.0	2	0.95
06XX	05XX	03XX	44.0mod			

SLA, Swine Leucocyte Antigen; Lr-Hp, Low resolution Haplotype; GT, Genotype; No’s, number of animals; Freq, frequency (in %); GMP, Göttingen Minipig; YDLR, Yorkshire/Duroc/Landrace 3-way crossbreed; mod, modified; Blank, Indicating alleles that cannot be identified with the study primer sets. Preferred allele or allele group is underlined.

### 3.3 SLA class II diversity in Göttingen Minipigs

SLA class II genotypes were assigned with the same interpretation approach as for the SLA class I genotypes. The list of genotypes and haplotypes for all studied animals found in this metadata analysis is provided in **Supplementary 7**. The evaluation of each of the 209 digitized gel images revealed seven different genotypes occurring in the six cohorts of Göttingen Miniature pigs (22–24). In 209 Göttingen Minipigs, we found 7 distinct SLA class II genotypes. Assigned genotypes with corresponding allele groups/and or alleles and SLA class II low-resolution haplotypes of the investigated animals are displayed in **Table 2**. Every investigated cohort had a minimum of three different genotypes. The cohort 2021 exhibited the maximum of six different defined genotypes. The most common genotype GT-0.5 occurred in all six minipig cohorts and was documented in 67 animals at a frequency of 32.05% (**Table 2**; **Supplementary Figure 2**). This genotype comprises Lr-0.03 in combination with Lr-0.21 (**Table 2**). The genotype GT-0.6 was also distributed among all cohorts, with a total of 58 animals positive for this genotype (frequency: 27.75%). Thirty out of 209 minipigs were positive for SLA class II genotype with assigned number GT-0.4 that carries Lr-0.17 in combination with Lr-0.21 and Lr-0.17mod with Lr-0.21. This haplotype combination was found in five out of six cohorts, except cohort 2016, at a frequency of 14.35% (**Table 2**; **Supplementary Figure 2**). Genotype GT-0.3 was present in 28 minipigs from all six cohorts. Genotype GT-0.1 was present in 16 minipigs and is homozygous Lr-0.03 (frequency: 8.61%). Genotypes GT-0.2 and GT-0.7 were present in 5 minipigs each, at a frequency of 2.39% (**Table 2**; **Supplementary Figure 2**). Interestingly, genotype GT-0.2 is homozygous for Lr-0.17mod. The depicted allele groups gave rise to five class II haplotypes with Lr-0.21 [DRB1*01XX-DQB1*05XX-DQA*04XX(+05XX)] being the most frequent haplotype (38.28%) followed by Lr-Hp 0.03 [DRB1*03XX(03:02)-DQB1*03XX(03:01)-DQA*01XX] with 30.38%, as depicted in **Supplementary Table 4**; **Figure 2**. Lr-0.17 and Lr-Pie-0.1 occurred with a frequency of 16.27% and 13.88%, respectively. Lr-0.31 explained only 1.20% of the diversity of all cohorts.

**Table 2:** SLA class II genotypes and haplotypes found in 209 Göttingen Minipigs.

DRB1	DQB1	DQA	Lr-Hp	GT	No’s	Frq(%)
03XX(03:02)	03XX(03:01)	01XX	0.03	0.1	16	8.61
03XX(03:02)	03XX(03:01)	01XX	0.03			
08XX	05XX	04XX(+05XX)	0.17mod	0.2	5	2.39
08XX	05XX	04XX(+05XX)	0.17mod			
03XX(03:02)	03XX(03:01)	01XX	0.03	0.3	28	12.44
08XX	05XX	04XX(+05XX)	0.17mod			
08XX	05XX	Blank or Null	0.17	0.4	30	14.35
01XX	05XX	04XX(+05XX)	0.21			
08XX	05XX	04XX(+05XX)	0.17mod			
01XX	05XX	04XX(+05XX)	0.21			
03XX(03:02)	03XX(03:01)	01XX	0.03	0.5	67	32.05
01XX	05XX	04XX(+05XX)	0.21			
01XX	05XX	04XX(+05XX)	0.21	0.6	58	27.75
01XX	05XX	Blank	Pie-0.1			
01XX	05XX	04XX(+05XX)	0.21	0.7	5	2.39
03XX	05XX	04XX(+05XX)	0.31			

SLA, Swine Leucocyte Antigen; Lr-Hp, Low resolution Haplotype; GT, Genotype; No’s, number of animals; Freq, frequency (in %); mod, modified; Blank, Indicating alleles that cannot be identified with the study primer sets; mod, modified; Pie, Pietrain.

### 3.4 Genotypes and haplotypes for all studied Göttingen Minipigs

Among 209 studied Göttingen Minipigs, 60 different genotype combinations were found. Number and frequency of found genotype combinations are summarized in **Supplementary Table 5**. The graphical representation of the different combination genotypes in all 209 Minipigs can be visualized in **Figure 1**. The most common genotype combination was GT-3.0 with GT-0.4 present in 18 out of 209 Göttingen Minipigs corresponding to a frequency of 8.61% (**Supplementary Table 5**; **Figure 1**). The genotype combinations GT-1.0/GT-0.6 and GT-2.0/GT-0.5 occurred at frequencies of 8.13 and 7.66%, respectively. All SLA class I and SLA class II genotypes and haplotypes found in 209 studied Göttingen Minipigs are summarized in **Supplementary Table 6**.

### 3.5 Frequencies of found SLA class I and class II allele groups

The most frequent allele group in SLA-1 was 05XX/15XX occurring at 23.68% (**Table 3**; **Supplementary Figure 3**). The second most frequent allele group was 16:02(16:03) with a frequency of 18.90%. Allele group 08XX occurred at 15.55% and allele group 15XX at 14.83%, respectively. The frequencies of allele group 05XX was 10.77% and for 06XX and 04XX the frequency was 0.48% respectively. Blank alleles occurred at a frequency of 12.92% and null alleles with a frequency of 2.39%. For SLA-3, the most frequent was allele group 04XX with 29.43% and the lowest was 03XX with a 6.94% frequency (**Table 3**; **Supplementary Figure 3**). For SLA-2, the most frequent allele was 06XX with 30.62% and the lowest allele group was 04XX with a frequency of 0.24% (**Table 3**; **Supplementary Figure 3**). Allele group 01XX and 03XX occurred at the same frequency of 17.22%. The most frequent allele group in DRB1 group was 01XX occurring at 52.15% (**Table 3**; **Supplementary Figure 3**). The frequency of the second most frequent allele group was 03XX(03:02) with a frequency of 31.58%. Allele group 08XX occurred at 16.27%. For DQB1, allele group 5XX was the most frequent with 69.62% and 03XX(03:01) the second most frequent with 30.38% (**Table 3**; **Supplementary Figure 3**). For DQA, 04XX+05XX was the most frequent with 51.44%, followed by 01XX with 30.86% and Blank allele group with 13.40%. Null allele group had the lowest frequency with 4.31% (**Table 3**; **Supplementary Figure 3**).

**Table 3:** Allele groups frequencies for the studied 209 Göttingen Minipigs.

	Allele group	Frq (%)		Allele group	Frq (%)
**SLA-1**	04XX	0.48	**SLA-2**	01XX	17.22
	05XX	10.77		03XX	17.22
	06XX	0.48		04XX	0.24
	08XX	15.55		06XX	24.64
	05XX/15XX	23.68		08XX	0.24
	15XX	14.83		11:04	5.5
	16:02(16:03)	18.9		01XX/03XX	1.67
	Blank	12.92		03XX/17:01	18.66
	Null	2.39		Blank	8.61
**SLA-3**	03XX	6.94	**DRB1**	01XX	52.15
	04XX	29.43		03XX(03:02)	31.58
	05XX	22.25		08XX	16.27
	08XX	12.44	**DQB1**	03XX(03:01)	30.38
	03XX(03:04)/08XX	18.66		05XX	69.62
	05XX/08XX	10.29	**DQA**	01XX	30.86
				04XX(+05XX)	51.44
				Blank	13.4
				Blank or Null	4.31

SLA, Swine Leucocyte Antigen; Blank, Indicating alleles that cannot be identified with the study primer sets; Freq, frequency (in %). Preferred allele or allele group is underlined.

### 3.6 Comparative analysis with European farmed pigs

**Figure 3:**
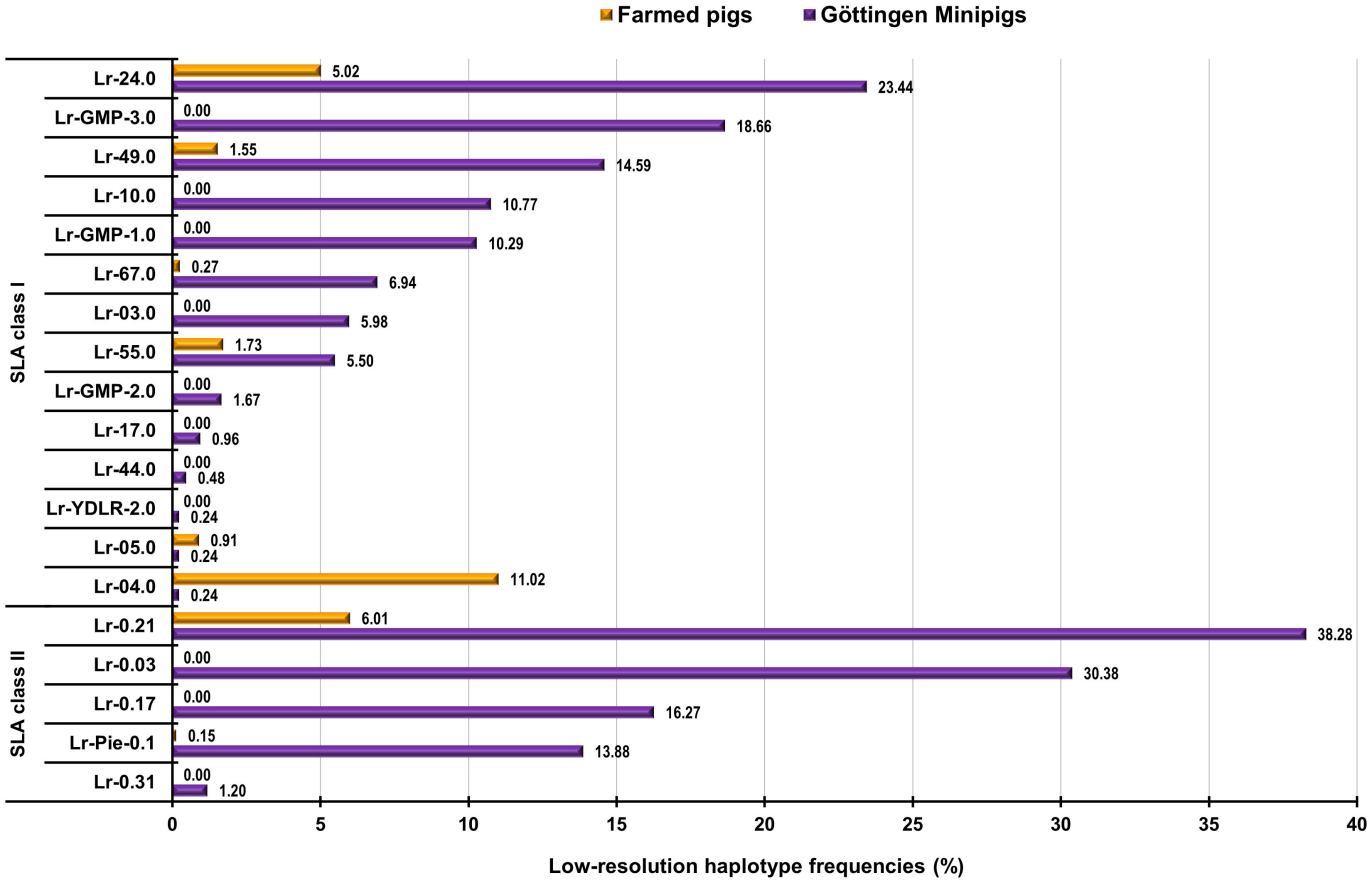
Graphical representation of frequencies of shared SLA class I and class II haplotypes in Göttingen Minipigs and European farmed pigs. SLA, Swine Leucocyte Antigen; Lr, Low-resolution Haplotype; GMP, Göttingen Minipig; YDLR, Yorkshire/Duroc/Landrace 3-way crossbreed; Pie, Pietrain. (29).

**Figure 4:**
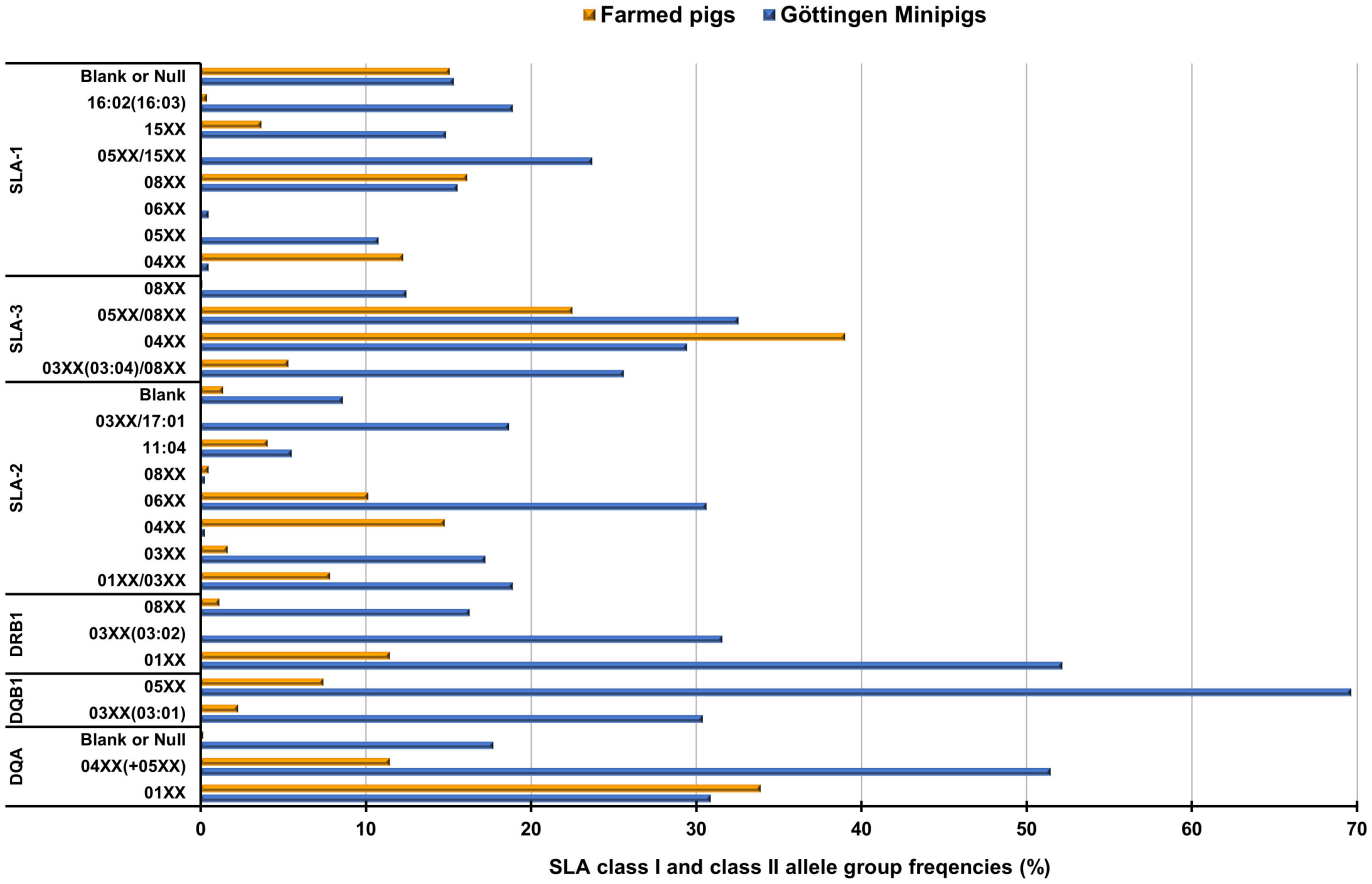
Graphical representation of frequencies of shared SLA class I and class II allele groups in Göttingen Minipigs and European farmed pigs. For detailed percentages please refer to **Supplementary Table 8**. SLA, Swine Leucocyte Antigen; Blank = Indicating alleles that cannot be identified with the study primer sets (29).

We compared the findings regarding the obtained haplotypes in the animals comprising this study with previously SLA-typing studies in European farmed pigs (29). Shared haplotypes and their frequencies between Göttingen Minipigs and European farmed pigs are detailed in **Supplementary Table 7** and can be visualized in **Figure 3**. SLA class I haplotype Lr-Hp 24.0 which occurred with a frequency of 23.44% in Minipigs was also present in the farmed pigs with a frequency of 5.02% (**Supplementary Table 7**; **Figure 3**). Haplotypes Lr-Hp GMP-3.0 occurred in the Minipigs population at a frequency of 18.66% but was absent in the farmed pig populations. Haplotype Lr-Hp 49.0 was present in both populations with a frequency of 14.59% for the Minipigs and 1.55% for the farmed pigs. Lr-Hp 55.0 and 67.0 were shared by both the Minipigs and farmed pigs at frequencies of 5.50% and 6.94% for the Minipigs and 1.83% and 0.29% respectively for the farmed pigs. In contrast, Lr-Hp 03.0, Lr-Hp 10.0, Lr-Hp 17.0, Lr-Hp 44.0, Lr-Hp GMP-1.0, Lr-Hp GMP-2.0, Lr-Hp GMP-3.0 and Lr-Hp YDLR-2.0 were only observed in the Minipigs (**Supplementary Table 7**; **Figure 3**). Shared allele groups and their frequencies between Göttingen Minipigs and farmed pigs are detailed in **Supplementary Table 8** and can be visualized in **Figure 4**. Although, Göttingen Minipigs and farmed pigs shared 5 out of 8 SLA-1 allele groups, the predominant allele group SLA-1*05XX/08XX has not been detected in European farmed pigs. All four SLA-3 allele groups were found in both populations and the most frequent allele group in Göttingen Minipigs SLA-3*-05XX/08XX was detected in farmed pigs at a frequency of 22.49%. For SLA-2, 7 out of 8 allele groups contribute to the SLA background in both cohorts. Interestingly, the most common allele group for Göttingen Minipigs SLA-2*06XX occurred in these animals at a much higher frequency than in European farmed pigs (30.62 vs. 10.14%) (**Supplementary Table 8**; **Figure 4**). Although, Göttingen Minipigs and farmed pigs shared 2 out of 3 DRB1 allele groups, the predominant allele group DRB1*01XX has been detected in farmed pigs at a much lower frequency (52.15 vs. 11.45%). Both DQB1 allele groups were found in both populations and again, the most frequent allele group in Göttingen Minipigs DQB1*05XX was detected in farmed pigs at a frequency of only 7.42%. For DQA, all three allele groups contribute to the SLA background in both cohorts. Remarkably, the most common allele group for Göttingen Minipigs DQA*04XX(+05XX) occurred in these animals at a much higher frequency than in European farmed pigs (51.44 vs. 11.45%) (**Supplementary Table 8**; **Figure 4**).

## 4 Discussion

### 4.1 SLA diversity in Göttingen Minipigs

As genetic variety is mandatory for populations to confront environmental development and transformation, the Swine Leucocyte Antigens (SLA) play a key role in initiating immune response. The genetic diversity of a population is expressed by detecting and predicting the allelic variation, level of polymorphism and heterozygosity for certain loci. This study represents the first comparative analysis of the SLA gene diversity in Göttingen Minipigs and farmed pig lines by low-resolution typing. The PCR-based typing approach has proven to be an accessible and reliable method to resolve the molecular character of the porcine MHC. By applying the PCR-SSP assay, we were able to identify distinct haplotypes and genotypes in a total cohort of 209 Göttingen Minipigs. This metadata analysis revealed 26 distinct SLA class I and seven different SLA class II genotypes comprising 14 and five different low-resolution haplotypes, respectively (22–24). The most abundant SLA class I haplotypes Lr-Hp 24.0mod (SLA-1*15XX-SLA-3*04:04-SLA-2*06:01~02) and Lr-Hp GMP-3.0 (SLA-1*16:02-SLA-3*03:04-SLA-2*17:01) occurred at frequency of 23.44% and 18.66%, respectively. The Lr-Hp 24.0mod haplotype could be derived from Lr-Hp 24.0 in which the allele group for SLA-1 was determined as ‘blank’, meaning it is still unknown. It can be speculated that the novel identified haplotype Lr-GMP-3.0 could be the same as X.0 being found in a cohort of 19 NGS-typed Göttingen Minipigs (36). The SLA class II haplotype Lr-Hp 0.21 was found at a frequency of 38.28% and is composed of DRB1*01XX/DQB1*05XX/DQA*04XX. Comparing these results with a study conducted in European commercial pig lines, this haplotype occurred in 41 out of 341 animals, resembling a frequency of 6.01% (29). In this study, we found 26 SLA class I genotypes but only seven for SLA class II. The most frequent genotype was GT-3.0 with a frequency of 11.96% and GT-1.0 with a frequency of 11.00%. For SLA class II, the most frequent genotype was GT-0.5 with a frequency of 32.05% and GT-0.6 with a frequency of 27.75%, respectively. Comparing these results with published data from Techakriengkrai and co-workers, we can speculate that the unknown haplotype SLA-1*Blank/SLA-3*04XX/SLA-2*11XX might be the same as Lr-55.0mod found in the present study (33). As this haplotype occurred in only one animal, this needs to be further investigated. Although, one newly suggested haplotype from a study being conducted by Sørensen and co-workers, Hr-Z.0 (SLA-1*22:01/SLA-3*03:01/SLA-2*03:01) could represent a modification of Lr-3.0, this haplotype was not found in our studied cohort of Göttingen Minipigs (36). Based upon the low variety of haplotypes together with their similarity and considering the genetic background of these animals, it can be speculated that present Göttingen Minipigs may already have a restricted gene pool. However, among the studied cohorts of Göttingen Minipigs, a couple of potential private haplotypes were found that may be specific only to these Minipigs. Commercial pigs exhibit a high SLA diversity being resembled in 91 SLA class I and 47 SLA class II haplotypes that have been so far identified in various pig lines (27–29, 32, 33, 36, 37). This assumption is supported by the restricted occurrence of the SLA class I haplotype Lr-Hp 10.0 and the SLA class II haplotypes Lr-Hp 0.3, Lr-Hp 0.17 and Lr-Hp 0.31mod being exclusively found in Göttingen Minipigs. Private haplotypes like GMP-1.0, GMP-2.0 and GMP-3.0 could also represent novel and private haplotypes in Göttingen Minipigs.

### 4.2 Shared SLA diversity with outbred pigs and miniature swine models

In European farmed pig breeds a wide range of haplotypes were characterized comprising of 91 SLA class I haplotypes and 47 SLA class II respectively (27–29, 32, 33, 36, 37). In Göttingen Minipigs, 14 SLA class I and 5 SLA class II haplotypes were assigned. In European commercial pig lines, Lr-Hp 24.0 was found in 55 out of 549 pigs, corresponding to a frequency of 5.01%. In Göttingen Minipigs, this haplotype was the most abundant one with a frequency of 23.44%. The second most frequent haplotype in Göttingen Minipigs Lr-GMP-3.0 with a frequency of 18.66% was not present in the farmed pig populations (29). Additionally, 22 Göttingen Minipigs were homozygous for either of the SLA class I haplotypes Lr-Hp 03.0, 24.0, 49.0 or GMP-3.0, whereas 21 animals were homozygous for SLA class II by carrying either Lr-Hp 0.03 or 0.17mod, which was not the case for the farmed pigs. The obtained data revealed that Göttingen Minipigs only share six SLA class I and two SLA class II haplotypes with European farmed pigs. More importantly, despite the limited number of SLA class I haplotypes, the high genotype diversity being observed necessitates pre-experimental SLA background assessment of Göttingen Minipigs in regenerative medicine and xenograft research. Although abundant haplotypes were established in farmed pig lines this could lead to reduced diversity over time and susceptibility to diseases. Regarding the Göttingen Minipigs, there is still the need for data based on larger cohorts of pigs and more extensive typing to explore their diversity. Around the world, SLA-inbred/-defined minipig lines have been established, including the National Institutes of Health/Massachusetts General Hospital (NIH/MGH) miniature swine model (38), Yucatan miniature pigs (39), Japanese Microminipigs (40), CLAWN miniature swine (41), British Babraham pigs (42), and Chinese Rongshui miniature pigs (37). In comparison with Göttingen Minipigs, NIH/MGH pigs share two SLA class I haplotypes, namely Hp-03.0 and Hp-04.0, the latter one was also found in Yucatan miniature pigs together with Hp-05.0, also being shared by Chinese Rongshui pigs. Japanese Microminipigs harbor the two shared SLA class I haplotypes Hp-10.0 and Hp-17.0, the latter haplotype was also observed in CLAWN miniature swine. Lastly, SLA-inbred British Babraham pigs share their SLA class I haplotype Hp-55.0 with Göttingen Minipigs. With respect to SLA class II haplotypes, only two haplotypes are also present in SLA-inbred/-defined minipig lines. NIH/MGH pigs share Hp-0.03, whereas Japanese Microminipigs and CLAWN pigs have Hp-0.17 in common with Göttingen Minipigs.

### 4.3 Confirmatory potential of the PCR-based typing approach

For investigation of occurring SLA alleles and allele groups in the animals that make the subject of this project, low-resolution typing using SSP-PCR represents a convenient and relatively accessible strategy for the molecular characterization of the porcine MHC. With several different primer combinations this technique allows an examination of the presence of various alleles and allele groups leading to a more detailed picture summarizing occurring SLA class I and class II genes. This method elucidated the SLA region of the porcine genome and enabled the recognition of appearing alleles in various pig breeds (27–29, 33, 37, 43–45). However, this approach has some weak points as it is highly dependent on the selection of used primers and may generate inaccurate and occasionally insufficient results. As an alternative, sequence-based typing (SBT) strategy offers a thorough analysis of such misinterpretation and determines the alleles that could not be specifically identified by low-resolution typing. This method is time-consuming as it may include cloning and sequencing, but for the resulting accuracy the SBT approach may be an exceptionally useful tool for molecular characterization of unknown alleles. However, successful translation of NGS-based approaches will help to facilitated SBT in pig populations being using in biomedical research (36, reviewed in 19). The results of such typing are mostly explicit and contribute to detection and description of specific sequences. Thus, high-resolution typing presents an effective **Supplementary Method** in case of insufficient data acquired by low-resolution typing.

### 4.4 Implications of the SLA background of Göttingen Minipigs for its role as an animal model in biomedical research

Developing suitable animal models is a crucial prerequisite for the development of safe preclinical protocols in biomedical research. Miniature pigs can be used in long-term experiments owing to their long lifespan; they can also be easily bred and handled because of their small size and short reproduction cycle (8). Miniature pigs have become promising donor animals for xenotransplantation because of their anatomical and physiological characteristics that are very similar to those of humans (4, 5, 16). Similarly, to allogeneic transplantation, the high MHC variability also represents a major immunological hurdle in xenotransplantation as it allows for the presentation of the genetic differences between donor and recipient at major and minor histocompatibility antigen level (46–48). Incompatible MHC genes inflicts an acute cellular rejection [immunological rejection including hyperacute rejection (HAR), acute humoral xenograft rejection (AHXR) or immune cell-mediated rejection] to the recipient leading to immediate graft loss or development of a chronic rejection that induces, in most cases, a late graft failure (49, 50). Biomedical research is working towards unraveling the enormous impact that MHC has on the response of a human body to a received transplant and understanding the MHC function and immunologically initiated processes could eventually represent an absolute solution to these complications (19, 51, 52). In this matter, the use of Göttingen Minipigs with defined genetically background that have distinguished significant similarities with the human body and its physiological, immunological, and pathological mechanisms, could be highly beneficial in transplant studies soon.

## 5 Conclusions

In this project, we conducted a metadata analysis of low-resolution PCR-SSP typing results from a cohort of 209 Göttingen Minipigs deriving from the populations being housed at the company Ellegaard Göttingen Minipigs A/S. Considering the genetically background of these animals and the similarity and low variety of haplotypes, we can argue that Göttingen Minipigs have a restricted gene pool at least for their MHC loci. Seven of the haplotypes being observed in the Göttingen Minipigs were found in various populations of farmed pig. This should be considered before using Göttingen Minipigs as a model for vaccine development studies. Furthermore, the Göttingen Minipigs displayed the highest percentage of homozygous individuals. This might be advantageous, e.g., for uniform responses in immunogenicity studies, but not representative for more outbred pig breeds. The found genotypes and haplotypes together with their frequencies and their resemblance point towards a restricted SLA diversity in this pig breed, which could be a limiting factor in later mismatch donor allotransplant studies. Overall, the large variation in haplotypes between distinct pig populations highlights the importance of SLA typing when designing vaccines for worldwide use.

## Data availability statement

The datasets presented in this study can be found in online repositories. The names of the repository/repositories and accession number(s) can be found in the article/**Supplementary Material**.

## Ethics statement

The animal study was approved by Austrian Federal Ministry of Education, of Science and Research. The study was conducted in accordance with the local legislation and institutional requirements.

## Author contributions

SH: Conceptualization, Project administration, Visualization, Writing – original draft. TD: Formal analysis, Methodology, Writing – review & editing. MG: Formal analysis, Methodology, Writing – original draft. SG: Formal analysis, Methodology, Writing – review & editing. CP: Methodology, Resources, Writing – review & editing. KH: Project administration, Resources, Writing – review & editing. LK: Formal analysis, Methodology, Writing – review & editing. JS: Formal analysis, Methodology, Writing – review & editing. MS: Formal analysis, Methodology, Writing – review & editing. AJ: Resources, Writing – review & editing. AS: Resources, Supervision, Writing – review & editing. NW: Project administration, Resources, Writing – review & editing. CF: Funding acquisition, Project administration, Writing – review & editing.
