# Supplementary material for: Comparative analysis of swine leukocyte antigen gene diversity in Göttingen Minipigs
Source: Front Immunol. 2024 Feb 26;15:1360022. doi: 10.3389/fimmu.2024.1360022 (PMC10925748; doi:10.3389/fimmu.2024.1360022)
Supplement: Supplementary file 1 [file DataSheet_1.docx]

Supplementary Material

**Comparative analysis of swine leukocyte antigen (SLA) gene diversity in Göttingen Minipigs**

**Sabine E. Hammer^1,*^, Tereza Duckova^1^, Monica Gociman^1^, Sandra Groiss^1^, Clara P.S. Pernold^1^, Karolin Hacker^2^, Lena Kasper^3^, Julia Sprung^1^, Maria Stadler^1^, Andres Eskjær Jensen^4^, Armin Saalmüller^1^, Nadine Wenzel^2^, Constanca Figueiredo^2^**

^1^Institute of Immunology, Department of Pathobiology, University of Veterinary Medicine Vienna, Vienna, Austria

^2^Institute of Transfusion Medicine and Transplant Engineering, Hannover Medical School, 30625 Hannover, Germany

^3^Merck Healthcare KGaA, 64293 Darmstadt, Germany

^4^Ellegaard Göttingen Minipigs A/S, Dalmose, Denmark

*** Correspondence:**Sabine E. Hammer
sabine.hammer@vetmeduni.ac.at

Keywords: *Sus scrofa*, swine leukocyte antigen (SLA), polymorphism, animal model, biomedical research and development, transplantation, xenograft

# Funding

This research was funded by Deutsche Forschungsgemeinschaft (DFG) - Transregional Collaborative Research Centre 127 (TRR127: Biology of xenogeneic cells, tissue and organ transplantation - from bench to bedside).

# Conflict of Interest

The authors declare that the research was conducted in the absence of any commercial or financial relationships that could be construed as a potential conflict of interest.

# Acknowledgments

We are thankful for the excellent technical assistance to Yvonne Speidel (Institute of Transfusion Medicine and Transplant Engineering, Hannover Medical School).

# Supplementary Figures and Tables

## Supplementary Figures


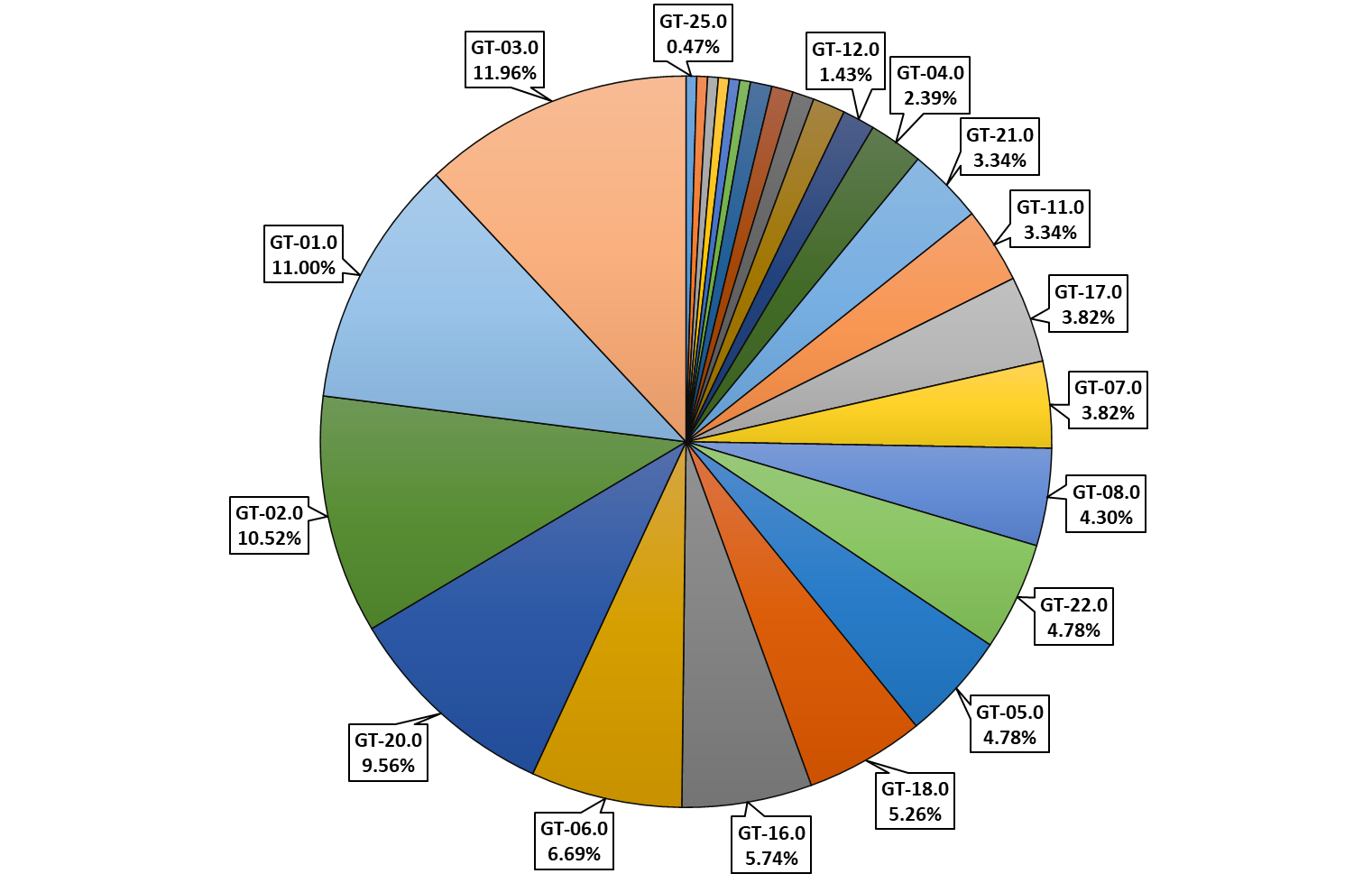


**Supplementary Figure 1.** Graphical representation of frequencies of found SLA class I genotypes in 209 Göttingen Minipigs. SLA = Swine leucocyte Antigen; GT = Genotype.


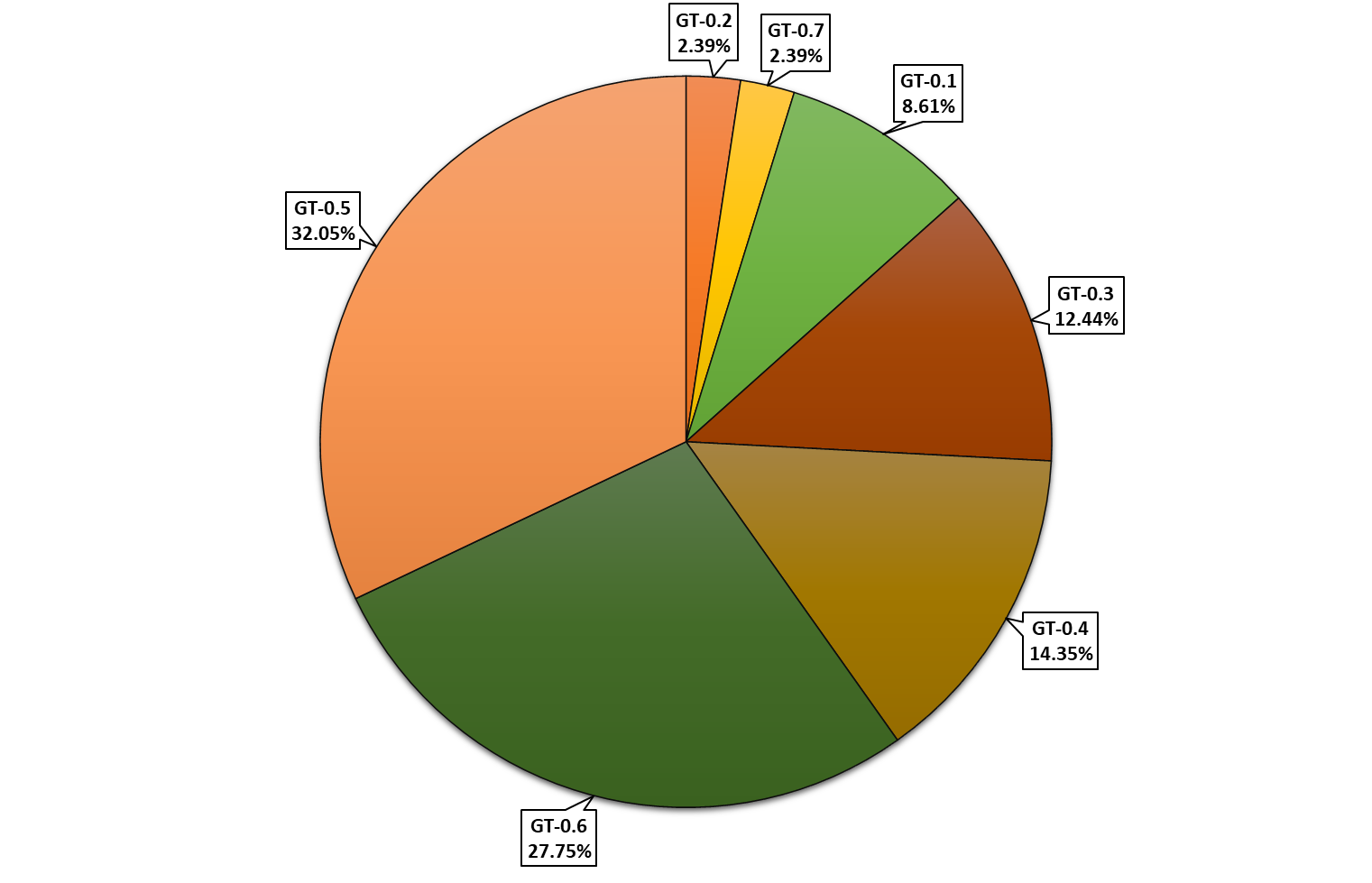


**Supplementary Figure 2.** Graphical representation of frequencies of found SLA class II genotypes in 209 Göttingen Minipigs. SLA = Swine leucocyte Antigen; GT = Genotype.

A

B

**Supplementary Figure 3.** Graphical representation of frequencies of found SLA class I (A) and class II (B) allele group frequencies in 209 Göttingen Minipigs. SLA = Swine Leucocyte Antigen; Blank = Indicating alleles that cannot be identified with the study primer sets.

## Supplementary Tables

**Supplementary Table 2.** Studied animals listed by cohort of origin

| **Cohort 2016** | **Cohort 2017** | **Cohort 2018** | | **Cohort 2019** | | | | | **Cohort 2021** | | **Cohort 2023** | | |
| --- | --- | --- | --- | --- | --- | --- | --- | --- | --- | --- | --- | --- | --- |
| ELLG 01 | 327335 | 229867 | 334795 | 335829 | 232510 | 337540 | 234131 | 333637 | | 333645 | 500299 | 244484 | 349928 |
| ELLG 02 | 326690 | 229886 | 335049 | 232336 | 233115 | 337476 | 234230 | 333711 | | 333674 | 500270 | 244319 | 351607 |
| ELLG 03 | 327458 | 332467 | 334816 | 335840 | 233058 | 234364 | 234164 | 333608 | | 333712 | 500370 | 244473 | 353100 |
| ELLG 04 | 226115 | 332528 | 231771 | 232337 | 337458 | 234163 | 333304 | 333641 | | 333722 | 500378 | 243595 | 351146 |
| ELLG 05 | 330792 | 332848 | 231454 | 335943 | 233061 | 339148 | 333504 | 333713 | | 333749 | 500363 | 243405 | 351483 |
| ELLG 06 | 330805 | 230014 | 334823 | 232474 | 337251 | 338853 | 335055 | 333692 | | 339280 | 244131 | 243809 | 351690 |
| ELLG 07 | 228577 | 230084 | 231604 | 335910 | 337442 | 338759 | 340259 | 333669 | | 339145 | 244980 | 244178 | 352478 |
| ELLG 08 | 228842 | 332561 | 231383 | 232438 | 337535 | 234140 | 340246 | 333745 | | 4163 | 244251 | 243829 | 352149 |
| ELLG 09 | 330745 | 332570 | 334694 | 336113 | 337110 | 234361 | 340255 | 333686 | | 4174 | 243893 | 244336 | 353087 |
| ELLG 10 | 228935 | 229930 | 334637 | 232427 | 337071 | 338800 | 340221 | 333615 | | 4170 | 244961 | 351486 | 351853 |
| ELLG 11 | 228482 | 230057 | 231615 | 335836 | 233163 | 234375 | 340219 | 228504 | | 4184 | 243855 | 351771 | 352069 |
| ELLG 12 | 330919 | 332516 | 231627 | 232455 | 233078 | 338825 | 340257 | 329952 | | 4180 | 244631 | 352290 |  |
| 6456 | 228518 | 332767 | 334800 | 335823 | 233292 | 338912 | 340253 | 330041 | | 4191 | 243911 | 351513 |  |
| 6461 | 330590 | 332387 |  | 232432 | 337215 | 338845 | 340247 | 234341 | | 4169 | 243641 | 352473 |  |
| 6462 | 330477 | 231672 |  | 336141 | 233057 | 234121 | 340226 | 231975 | | 4177 | 244058 | 352498 |  |
| 6468 | 228838 | 231472 |  | 232499 | 337508 | 234089 | 340222 | 232107 | | 4182 | 243973 | 351120 |  |
| 6473 |  | 334653 |  | 336237 | 233184 | 234202 |  | 232004 | | 4185 | 244362 | 350675 |  |

ELLG = Ellegaard.

**Supplementary Table 2.** Genomic DNA concentrations and quality measures of the examined 209 Göttingen Minipigs

| **Cohort 2016** | | | |  | **Cohort 2018** | | | | | |
| --- | --- | --- | --- | --- | --- | --- | --- | --- | --- | --- |
| Sample ID | gDNA [ng/µl] | 260/280 | 260/230 |  | Sample ID | gDNA [ng/µl] | 260/280 | | 260/230 | |
| ELLG 01 | 53.00 | 1.96 | 1.83 |  | 229867 | 211.60 | 1.94 | | 2.23 | |
| ELLG 02 | 36.05 | 1.96 | 1.69 |  | 229886 | 246.60 | 1.93 | | 2.19 | |
| ELLG 03 | 42.75 | 1.91 | 1.47 |  | 230014 | 119.90 | 1.95 | | 2.11 | |
| ELLG 04 | 35.45 | 2.02 | 2.06 |  | 332387 | 203.15 | 1.93 | | 2.21 | |
| ELLG 05 | 31.55 | 1.98 | 2.26 |  | 332467 | 149.55 | 1.95 | | 2.17 | |
| ELLG 06 | 44.05 | 1.98 | 2.12 |  | 332528 | 139.80 | 1.94 | | 2.21 | |
| ELLG 07 | 47.50 | 1.96 | 2.24 |  | 332848 | 200.55 | 1.94 | | 2.14 | |
| ELLG 08 | 49.70 | 1.96 | 1.84 |  | 229930 | 168.40 | 1.95 | | 2.14 | |
| ELLG 09 | 42.55 | 1.94 | 1.85 |  | 230057 | 156.45 | 1.95 | | 2.13 | |
| ELLG 10 | 55.35 | 1.94 | 2.08 |  | 230084 | 112.45 | 1.97 | | 2.04 | |
| ELLG 11 | 39.75 | 2.00 | 2.11 |  | 332516 | 136.50 | 1.96 | | 2.06 | |
| ELLG 12 | 47.90 | 1.96 | 2.38 |  | 332561 | 112.95 | 1.95 | | 2.10 | |
| 6456 | 62.40 | 1.96 | 1.69 |  | 332570 | 187.95 | 1.94 | | 2.13 | |
| 6461 | 83.60 | 1.69 | 2.40 |  | 332767 | 179.60 | 1.94 | | 2.10 | |
| 6462 | 88.05 | 1.93 | 1.70 |  | 231454 | 147.80 | 1.87 | | 2.30 | |
| 6468 | 92.40 | 1.94 | 1.81 |  | 231627 | 107.90 | 1.84 | | 2.18 | |
| 6473 | 96.90 | 1.93 | 2.50 |  | 231771 | 70.00 | 1.85 | | 2.32 | |
| **Cohort 2017** | | | |  | 334653 | 177.40 | | 1.88 | | 2.31 |
| Sample ID | gDNA [ng/µl] | 260/280 | 260/230 |  | 334694 | 271.35 | 1.87 | | 2.35 | |
| 326690 | 46.8 | 2.11 | 1.57 |  | 334795 | 115.75 | 1.88 | | 2.25 | |
| 226115 | 64.1 | 1.68 | 1.54 |  | 334800 | 162.95 | 1.87 | | 2.30 | |
| 327335 | 42.2 | 1.87 | 1.66 |  | 334816 | 100.35 | 1.87 | | 2.38 | |
| 327485 | 60.6 | 1.81 | 1.56 |  | 231383 | 137.15 | 1.86 | | 2.00 | |
| 330805 | 39.60 | 1.96 | 1.50 |  | 231472 | 92.20 | 1.90 | | 2.31 | |
| 228842 | 47.10 | 1.95 | 1.84 |  | 231604 | 104.95 | 1.90 | | 2.46 | |
| 228935 | 29.85 | 2.00 | 1.71 |  | 231615 | 117.60 | 1.88 | | 2.39 | |
| 330919 | 31.10 | 2.04 | 1.81 |  | 231672 | 113.30 | 1.91 | | 2.36 | |
| 330590 | 37.80 | 1.97 | 1.92 |  | 334637 | 127.40 | 1.87 | | 2.35 | |
| 228838 | 21.35 | 2.07 | 1.54 |  | 334823 | 153.15 | 1.89 | | 2.42 | |
| 330792 | 32.45 | 1.99 | 1.70 |  | 335049 | 141.40 | 1.91 | | 2.33 | |
| 228577 | 29.50 | 1.99 | 1.45 |  |  |  |  | |  | |
| 330745 | 19.05 | 2.10 | 1.55 |  |  |  |  | |  | |
| 228482 | 19.65 | 2.00 | 1.32 |  |  |  |  | |  | |
| 228518 | 32.50 | 1.97 | 1.54 |  |  |  |  | |  | |
| 330477 | 12.20 | 2.16 | 1.12 |  |  |  |  | |  | |

**Supplementary Table 2.** Genomic DNA concentrations and quality measures of the examined 209 Göttingen Minipigs (continued)

| **Cohort 2019** | | | |  | **Cohort 2019** | | | |
| --- | --- | --- | --- | --- | --- | --- | --- | --- |
| Sample ID | gDNA [ng/µl] | 260/280 | 260/230 |  | Sample ID | gDNA [ng/µl] | 260/280 | 260/230 |
| 335823 | 42.60 | 2.03 | 1.95 |  | 337442 | 45.20 | 2.08 | 2.10 |
| 335829 | 67.60 | 2.08 | 2.06 |  | 337476 | 90.65 | 2.06 | 2.07 |
| 335836 | 72.95 | 2.11 | 2.08 |  | 234121 | 48.75 | 2.02 | 1.64 |
| 335840 | 53.65 | 2.10 | 2.05 |  | 234202 | 14.63 | 2.22 | 1.04 |
| 335910 | 57.70 | 2.08 | 1.98 |  | 234230 | 39.75 | 2.03 | 1.73 |
| 335943 | 69.35 | 2.04 | 1.98 |  | 234361 | 26.90 | 2.11 | 1.39 |
| 336113 | 52.90 | 2.10 | 2.00 |  | 234364 | 32.33 | 2.08 | 1.34 |
| 336141 | 75.30 | 2.09 | 2.07 |  | 234375 | 35.20 | 2.03 | 1.41 |
| 336237 | 52.65 | 2.03 | 1.92 |  | 338759 | 24.20 | 2.12 | 1.87 |
| 232336 | 118.85 | 1.97 | 2.17 |  | 338912 | 26.47 | 2.13 | 1.41 |
| 232337 | 72.65 | 2.00 | 2.08 |  | 339148 | 22.30 | 2.10 | 1.49 |
| 232427 | 75.90 | 1.97 | 2.03 |  | 234089 | 25.70 | 1.96 | 0.83 |
| 232432 | 46.50 | 2.06 | 2.26 |  | 234131 | 22.05 | 2.15 | 1.73 |
| 232438 | 38.60 | 2.02 | 1.93 |  | 234140 | 30.50 | 2.04 | 1.18 |
| 232455 | 56.80 | 2.00 | 2.07 |  | 234163 | 19.85 | 2.24 | 1.27 |
| 232474 | 31.65 | 2.03 | 1.92 |  | 234164 | 21.95 | 2.14 | 1.31 |
| 232499 | 55.75 | 1.99 | 2.01 |  | 338800 | 23.77 | 2.09 | 1.20 |
| 232510 | 57.85 | 2.01 | 2.03 |  | 338825 | 26.60 | 1.98 | 1.48 |
| 233078 | 94.50 | 2.05 | 1.93 |  | 338845 | 19.73 | 1.96 | 1.06 |
| 233115 | 76.00 | 2.18 | 2.21 |  | 338853 | 24.10 | 1.98 | 0.76 |
| 337071 | 76.80 | 2.23 | 2.30 |  | 333304 | 158.25 | 1.92 | 2.21 |
| 337215 | 94.70 | 2.23 | 2.27 |  | 333504 | 111.25 | 1.93 | 2.27 |
| 337251 | 57.00 | 2.20 | 2.24 |  | 335055 | 109.75 | 1.92 | 2.26 |
| 337458 | 88.35 | 2.29 | 2.26 |  | 340259 | 127.50 | 2.02 | 2.34 |
| 337508 | 72.40 | 2.07 | 2.15 |  | 340246 | 88.70 | 1.99 | 2.41 |
| 337535 | 98.50 | 1.91 | 1.85 |  | 340255 | 68.80 | 1.90 | 1.81 |
| 337540 | 97.80 | 2.13 | 2.14 |  | 340221 | 62.90 | 1.96 | 2.37 |
| 233057 | 65.70 | 2.03 | 2.05 |  | 340219 | 72.55 | 1.94 | 2.41 |
| 233058 | 53.30 | 2.07 | 2.02 |  | 340257 | 57.95 | 1.97 | 2.40 |
| 233061 | 46.60 | 1.85 | 1.87 |  | 340253 | 51.65 | 2.06 | 2.25 |
| 233163 | 58.60 | 1.88 | 2.00 |  | 340247 | 60.65 | 1.96 | 2.00 |
| 233184 | 85.30 | 2.23 | 2.23 |  | 340226 | 53.80 | 2.01 | 2.15 |
| 233292 | 51.50 | 2.01 | 2.03 |  | 340222 | 122.65 | 2.03 | 2.26 |
| 337110 | 53.75 | 1.95 | 1.94 |  |  |  |  |  |

**Supplementary Table 2.** Genomic DNA concentrations and quality measures of the examined 209 Göttingen Minipigs (continued)

| **Cohort 2021** | | | |  | **Cohort 2021** | | | |
| --- | --- | --- | --- | --- | --- | --- | --- | --- |
| Sample ID | gDNA [ng/µl] | 260/280 | 260/230 |  | Sample ID | gDNA [ng/µl] | 260/280 | 260/230 |
| 333637 | 65.40 | 1.99 | 2.29 |  | 333645 | 26.57 | 2.02 | 1.55 |
| 333711 | 35.10 | 2.13 | 2.44 |  | 333674 | 101.67 | 1.94 | 2.08 |
| 333608 | 159.95 | 1.90 | 2.20 |  | 333712 | 296.75 | 1.90 | 2.17 |
| 333641 | 95.18 | 1.94 | 2.12 |  | 333722 | 75.34 | 1.95 | 2.02 |
| 333713 | 60.80 | 2.01 | 2.09 |  | 333749 | 286.63 | 1.89 | 2.23 |
| 333692 | 272.83 | 1.89 | 2.33 |  | 339280 | 283.10 | 1.90 | 2.20 |
| 333669 | 75.27 | 1.98 | 2.11 |  | 339145 | 405.61 | 1.90 | 2.28 |
| 333745 | 150.05 | 1.90 | 2.17 |  | 4163 | 56.55 | 2.02 | 3.39 |
| 333686 | 122.60 | 1.94 | 2.23 |  | 4174 | 46.85 | 2.01 | 2.85 |
| 333615 | 162.24 | 1.92 | 2.20 |  | 4170 | 50.70 | 1.99 | 3.11 |
| 228504 | 92.35 | 1.95 | 2.17 |  | 4184 | 24.35 | 2.09 | 3.36 |
| 329952 | 167.83 | 1.92 | 2.19 |  | 4180 | 23.30 | 2.08 | 1.87 |
| 330041 | 199.65 | 1.92 | 2.00 |  | 4191 | 65.15 | 2.00 | 2.78 |
| 234341 | 107.10 | 1.94 | 1.80 |  | 4169 | 44.05 | 2.03 | 3.55 |
| 231975 | 119.47 | 1.94 | 1.90 |  | 4177 | 16.70 | 2.17 | 4.10 |
| 232107 | 138.65 | 1.93 | 1.97 |  | 4182 | 60.80 | 2.06 | 2.75 |
| 232004 | 359.67 | 1.90 | 2.17 |  | 4185 | 35.40 | 2.10 | 4.02 |
| **Cohort 2023** | | | |  | **Cohort 2023** | | | |
| Sample ID | gDNA [ng/µl] | 260/280 | 260/230 |  | Sample ID | gDNA [ng/µl] | 260/280 | 260/230 |
| 500299 | 209.30 | 1.86 | 1.58 |  | 244484 | 328.20 | 1.87 | 1.87 |
| 500270 | 185.90 | 1.83 | 1.62 |  | 244319 | 223.30 | 1.86 | 1.56 |
| 500370 | 340.40 | 1.89 | 1.78 |  | 244473 | 294.00 | 1.86 | 1.74 |
| 500378 | 200.90 | 1.75 | 1.47 |  | 243595 | 125.30 | 1.86 | 1.41 |
| 500363 | 368.80 | 1.82 | 1.85 |  | 243405 | 159.00 | 1.84 | 1.38 |
| 244131 | 200.40 | 1.87 | 1.46 |  | 243809 | 166.30 | 1.78 | 1.45 |
| 244980 | 395.40 | 1.98 | 1.85 |  | 244178 | 177.60 | 1.86 | 1.60 |
| 244251 | 224.90 | 1.87 | 1.51 |  | 243829 | 124.70 | 1.85 | 1.25 |
| 243893 | 204.20 | 1.88 | 1.55 |  | 244336 | 225.90 | 1.88 | 1.64 |
| 244961 | 508.00 | 1.96 | 1.96 |  | 351486 | 132.10 | 1.84 | 1.39 |
| 243855 | 202.90 | 1.86 | 1.52 |  | 351771 | 313.50 | 1.84 | 1.85 |
| 244631 | 309.20 | 1.85 | 1.96 |  | 352290 | 214.70 | 1.90 | 1.58 |
| 243911 | 101.80 | 1.78 | 1.34 |  | 351513 | 110.50 | 1.84 | 1.37 |
| 243641 | 171.90 | 1.90 | 1.61 |  | 352473 | 170.70 | 1.82 | 1.39 |
| 244058 | 183.40 | 1.88 | 1.62 |  | 352498 | 206.90 | 1.89 | 1.64 |
| 243973 | 287.20 | 1.85 | 1.84 |  | 351120 | 213.90 | 1.86 | 1.65 |
| 244362 | 281.10 | 1.84 | 1.76 |  | 350675 | 137.10 | 1.80 | 1.37 |

**Supplementary Table 2.** Genomic DNA concentrations and quality measures of the examined 209 Göttingen Minipigs (continued)

| **Cohort 2023** | | | |  | **Cohort 2023** | | | |
| --- | --- | --- | --- | --- | --- | --- | --- | --- |
| Sample ID | gDNA [ng/µl] | 260/280 | 260/230 |  | Sample ID | gDNA [ng/µl] | 260/280 | 260/230 |
| 349928 | 179.10 | 1.89 | 1.51 |  | 352478 | 467.60 | 1.84 | 1.97 |
| 351607 | 153.20 | 1.85 | 1.55 |  | 352149 | 286.70 | 1.92 | 1.81 |
| 353100 | 230.70 | 1.86 | 1.84 |  | 353087 | 369.10 | 1.86 | 1.90 |
| 351146 | 171.60 | 1.87 | 1.71 |  | 351853 | 262.00 | 1.87 | 1.92 |
| 351483 | 183.30 | 1.85 | 1.71 |  | 352069 | 218.10 | 1.86 | 1.70 |
| 351690 | 135.50 | 1.87 | 1.56 |  | 352149 | 286.70 | 1.92 | 1.81 |

ELLG = Ellegaard; ID = identification number.

**Supplementary Table 3.** Frequencies of found SLA class I haplotypes in 209 Göttingen Minipigs

| **SLA-1** | **SLA-3** | **SLA-2** | **Lr-Hp** | **No's** | **No's** | **Frq(%)** |
| --- | --- | --- | --- | --- | --- | --- |
| Null | 03XX(03:01~05/03:08~09) | 03XX | 03.0 | **10** | **25** | **5.98** |
| 15XX | 03XX(03:01~05/03:08~09) | 03XX | 03.0mod | **15** |  |  |
| 04XX | 04XX | 04XX | 04.0 | **1** | **1** | **0.24** |
| 04XX | 05XX | 08XX | 05.0 | **1** | **1** | **0.24** |
| 05XX | 08XX | 03XX | 10.0 | **45** | **45** | **10.77** |
| 08XX(08:04) | 03XX(03:04) | 06XX(06:03) | 17.0 | **4** | **4** | **0.96** |
| Blank | 04XX/04:04 | 06XX(06:01~02) | 24.0 | **49** | **98** | **23.44** |
| 05XX/15XX | 04XX/04:04 | 06XX(06:01~02) | 24.0mod | **49** |  |  |
| 16:02(16:03) | 04XX/04:04 | 06XX(06:04) | YDLR-2.0 | **1** | **1** | **0.24** |
| 06XX | 05XX | 03XX | 44.0mod | **2** | **2** | **0.48** |
| 08XX | 05XX | Blank | 49.0 | **36** | **61** | **14.59** |
| 08XX | 05XX | 01XX or 06XX | 49.0mod | **25** |  |  |
| 15XX | 04XX/04:04 | 11:04 | 55.0 | **18** | **23** | **5.50** |
| Blank | 04XX/04:04 | 11:04 | 55.0mod | **5** |  |  |
| 15XX | 05XX | 01XX | 67.0mod | **29** | **29** | **6.94** |
| 05XX/15XX | 05XX/08XX | 01XX | GMP-1.0 | **43** | **43** | **10.29** |
| 05XX/15XX | 08XX | 01XX/03XX | GMP-2.0 | **7** | **7** | **1.67** |
| 16:02 | 03XX(03:04)/08XX | 03XX/17:01 | GMP-3.0 | **78** | **78** | **18.66** |
|  |  |  |  | **418** | **418** | **100.00** |

SLA = Swine Leucocyte Antigen; Lr-Hp = Low resolution Haplotype; No’s = number of animals; Frq = frequency; mod = modified; Blank = Indicating alleles that cannot be identified with the study primer sets; YDLR = Yorkshire/Duroc/Landrace 3-way crossbreed; GMP = Göttingen Minipig.

**Supplementary Table 4.** Frequencies of found SLA class II haplotypes in 209 Göttingen Minipigs

| **DRB1** | **DQB1** | **DQA** | **Lr-Hp** | **No's** | **No's** | **Frq(%)** |
| --- | --- | --- | --- | --- | --- | --- |
| 03XX(03:02) | 03XX(03:01) | 01XX | Lr-0.03 | **127** | **127** | **30.37** |
| 08XX | 05XX | Blank or Null | Lr-0.17 | **18** | **68** | **16.27** |
| 08XX | 05XX | 04XX(+05XX) | Lr-0.17mod | **50** |  |  |
| 01XX | 05XX | 04XX(+05XX) | Lr-0.21 | **160** | **160** | **38.28** |
| 01XX | 05XX | Blank | Lr-Pie-0.1 | **56** | **58** | **13.88** |
| 01XX | 05XX | 01XX | Lr-Pie-0.1mod | **2** |  |  |
| 03XX | 05XX | 04XX(+05XX) | Lr-0.31mod | **5** | **5** | **1.20** |
|  |  |  |  | **418** | **418** | **100.00** |

SLA = Swine Leucocyte Antigen; Lr-Hp = Low resolution Haplotype; No’s = number of animals; Frq = frequency; Blank = Indicating alleles that cannot be identified with the study primer sets; mod = modified; Pie = Pietrain.

**Supplementary Table 5.** Combined genotypes of all 209 studied Göttingen Minipigs

| **SLA-I GT** | **SLA-II GT** | **No's** | **Freq(%)** | **SLA-I GT** | **SLA-II GT** | **No's** | **Freq(%)** |
| --- | --- | --- | --- | --- | --- | --- | --- |
| 1 | 0.5 | 3 | 1.44 | 18 | 0.5 | **5** | **2.39** |
| 3 | 0.3 | 4 | 1.91 | 26 | 0.3 | **2** | **0.96** |
| 2 | 0.5 | 16 | 7.66 | 22 | 0.4 | **8** | **3.83** |
| 3 | 0.5 | 2 | 0.96 | 16 | 0.5 | **5** | **2.39** |
| 2 | 0.1 | 2 | 0.96 | 9 | 0.3 | **1** | **0.48** |
| 1 | 0.6 | 17 | 8.13 | 20 | 0.6 | **8** | **3.83** |
| 2 | 0.3 | 2 | 0.96 | 18 | 0.6 | **3** | **1.44** |
| 15 | 0.1 | 1 | 0.48 | 21 | 0.5 | **4** | **1.91** |
| 7 | 0.6 | 2 | 0.96 | 18 | 0.1 | **3** | **1.44** |
| 11 | 0.1 | 5 | 2.39 | 22 | 0.6 | **1** | **0.48** |
| 11 | 0.5 | 2 | 0.96 | 12 | 0.3 | **1** | **0.48** |
| 4 | 0.5 | 5 | 2.39 | 12 | 0.5 | **2** | **0.96** |
| 6 | 0.5 | 5 | 2.39 | 16 | 0.3 | **1** | **0.48** |
| 6 | 0.4 | 1 | 0.48 | 21 | 0.1 | **1** | **0.48** |
| 6 | 0.6 | 6 | 2.87 | 10 | 0.1 | **2** | **0.96** |
| 6 | 0.3 | 2 | 0.96 | 25 | 0.6 | **1** | **0.48** |
| 8 | 0.2 | 3 | 1.44 | 20 | 0.5 | **10** | **4.78** |
| 3 | 0.4 | 18 | 8.61 | 22 | 0.3 | **1** | **0.48** |
| 8 | 0.5 | 1 | 0.48 | 20 | 0.1 | **1** | **0.48** |
| 8 | 0.3 | 5 | 2.39 | 21 | 0.7 | **1** | **0.48** |
| 1 | 0.7 | 3 | 1.44 | 23 | 0.2 | **1** | **0.48** |
| 2 | 0.6 | 2 | 0.96 | 15 | 0.3 | **1** | **0.48** |
| 5 | 0.3 | 5 | 2.39 | 19 | 0.5 | **1** | **0.48** |
| 5 | 0.2 | 1 | 0.48 | 19 | 0.6 | **1** | **0.48** |
| 7 | 0.5 | 6 | 2.87 | 16 | 0.6 | **6** | **2.87** |
| 5 | 0.1 | 3 | 1.44 | 20 | 0.3 | **1** | **0.48** |
| 17 | 0.6 | 8 | 3.83 | 14 | 0.7 | **1** | **0.48** |
| 3 | 0.6 | 1 | 0.48 | 15 | 0.4 | **1** | **0.48** |
| 5 | 0.4 | 1 | 0.48 | 13 | 0.6 | **1** | **0.48** |
| 24 | 0.4 | 1 | 0.48 | 21 | 0.6 | **1** | **0.48** |
|  |  |  |  |  |  | **209** | **100.00** |

SLA = Swine Leucocyte Antigen; GT = Genotype; No’s = Numbers of animals; Freq = frequency (in %).

**Supplementary Table 6.** SLA-I and SLA-II genotypes and haplotypes of 209 Göttingen Minipigs

|  | **Pig ID** | **SLA-I** | **GT** | **SLA-II** | **GT** |  | **Pig ID** | **SLA-I** | **GT** | **SLA-II** | **GT** |
| --- | --- | --- | --- | --- | --- | --- | --- | --- | --- | --- | --- |
|  |  | **Lr-Hp** |  | **Lr-Hp** |  |  |  | **Lr-Hp** |  | **Lr-Hp** |  |
| **Cohort 2016** | ELLG 01 | 24.0mod | **1.0** | 0.21 | **0.5** | **Cohort 2017** | 327335 | 24.0 | **6.0** | 0.21 | **0.5** |
|  |  | GMP-1.0 |  | 0.03 |  |  |  | 55.0 |  | 0.03 |  |
|  | ELLG 02 | 24.0mod | **1.0** | 0.21 | **0.5** |  | 326690 | 24.0 | **6.0** | 0.21 | **0.4** |
|  |  | GMP-1.0 |  | 0.03 |  |  |  | 55.0 |  | 0.17mod |  |
|  | ELLG 03 | 24.0mod | **3.0** | 0.03 | **0.3** |  | 327458 | 24.0 | **6.0** | 0.21 | **0.6** |
|  |  | 49.0mod |  | 0.17mod |  |  |  | 55.0 |  | Pie-0.1 |  |
|  | ELLG 04 | 10.0 | **2.0** | 0.21 | **0.5** |  | 226115 | 24.0 | **6.0** | 0.17mod | **0.3** |
|  |  | 67.0mod |  | 0.03 |  |  |  | 55.0 |  | 0.03 |  |
|  | ELLG 05 | 24.0mod | **3.0** | 0.21 | **0.5** |  | 330792 | 49.0 | **8.0** | 0.17mod | **0.2** |
|  |  | 49.0mod |  | 0.03 |  |  |  | 55.0 |  | 0.17mod |  |
|  | ELLG 06 | 24.0mod | **1.0** | 0.21 | **0.5** |  | 330805 | 24.0 | **6.0** | 0.03 | **0.5** |
|  |  | GMP-1.0 |  | 0.03 |  |  |  | 55.0 |  | 0.21 |  |
|  | ELLG 07 | 10.0 | **2.0** | 0.21 | **0.5** |  | 228577 | 24.0 | **3.0** | 0.17mod | **0.4** |
|  |  | 67.0mod |  | 0.03 |  |  |  | 49.0 |  | 0.21 |  |
|  | ELLG 08 | 10.0 | **2.0** | 0.03 | **0.1** |  | 228842 | 49.0 | **8.0** | 0.03 | **0.5** |
|  |  | 67.0mod |  | 0.03 |  |  |  | 55.0 |  | 0.21 |  |
|  | ELLG 09 | 24.0mod | **1.0** | 0.21 | **0.6** |  | 330745 | 49.0 | **8.0** | 0.17mod | **0.2** |
|  |  | GMP-1.0 |  | Pie-0.1 |  |  |  | 55.0 |  | 0.17mod |  |
|  | ELLG 10 | 10.0 | **2.0** | 0.03 | **0.3** |  | 228935 | 24.0 | **6.0** | 0.21 | **0.6** |
|  |  | 67.0mod |  | 0.17mod |  |  |  | 55.0 |  | Pie-0.1 |  |
|  | ELLG 11 | 10.0 | **2.0** | 0.21 | **0.5** |  | 228482 | 24.0 | **6.0** | 0.03 | **0.5** |
|  |  | 67.0mod |  | 0.03 |  |  |  | 55.0 |  | 0.21 |  |
|  | ELLG 12 | 10.0 | **15.0** | 0.03 | **0.1** |  | 330919 | 49.0 | **8.0** | 0.17mod | **0.2** |
|  |  | 49.0mod |  | 0.03 |  |  |  | 55.0 |  | 0.17mod |  |
|  | 6456 | 10.0mod | **7.0** | 0.21 | **0.6** |  | 228518 | 24.0 | **6.0** | 0.21 | **0.6** |
|  |  | 24.0mod |  | Pie-0.1 |  |  |  | 55.0 |  | Pie-0.1 |  |
|  | 6461 | 24.0mod | **1.0** | 0.21 | **0.6** |  | 330590 | 49.0 | **3.0** | 0.17mod | **0.4** |
|  |  | GMP-1.0 |  | Pie-0.1 |  |  |  | 24.0 |  | 0.21 |  |
|  | 6462 | 03.0mod | **11.0** | 0.03 | **0.1** |  | 330477 | 24.0 | **3.0** | 0.17mod | **0.4** |
|  |  | 24.0 |  | 0.03 |  |  |  | 49.0 |  | 0.21 |  |
|  | 6468 | 03.0mod | **11.0** | 0.21 | **0.5** |  | 228838 | 24.0 | **6.0** | 0.21 | **0.6** |
|  |  | 24.0 |  | 0.03 |  |  |  | 55.0 |  | Pie-0.1 |  |
|  | 6473 | 24.0mod | **4.0** | 0.21 | **0.5** |  |  |  |  |  |  |
|  |  | GMP-2.0 |  | 0.03 |  |  |  |  |  |  |  |

**Supplementary Table 6.** SLA-I and SLA-II genotypes and haplotypes of 209 Göttingen Minipigs (continued)

|  | **Pig ID** | **SLA-I** | **GT** | **SLA-II** | **GT** |  | **Pig ID** | **SLA-I** | **GT** | **SLA-II** | **GT** |
| --- | --- | --- | --- | --- | --- | --- | --- | --- | --- | --- | --- |
|  |  | **Lr-Hp** |  | **Lr-Hp** |  |  |  | **Lr-Hp** |  | **Lr-Hp** |  |
| **Cohort 2018** | 229867 | 24.0 | **6.0** | 0.21 | **0.5** | **Cohort 2018** | 334653 | 10.0 | **2.0** | 0.21 | **0.5** |
|  |  | 55.0 |  | 0.03 |  |  |  | 67.0mod |  | 0.03 |  |
|  | 229886 | 24.0 | **6.0** | 0.21 | **0.5** |  | 334795 | 24.0mod | **4.0** | 0.21 | **0.5** |
|  |  | 55.0 |  | 0.03 |  |  |  | GMP-2.0 |  | 0.03 |  |
|  | 332467 | 24.0 | **6.0** | 0.17mod | **0.3** |  | 335049 | 24.0mod | **3.0** | 0.21 | **0.4** |
|  |  | 55.0 |  | 0.03 |  |  |  | 49.0mod |  | 0.17 |  |
|  | 332528 | 24.0 | **6.0** | 0.21 | **0.6** |  | 334816 | 24.0mod | **3.0** | 0.21 | **0.4** |
|  |  | 55.0 |  | Pie-0.1 |  |  |  | 49.0mod |  | 0.17 |  |
|  | 332848 | 24.0 | **6.0** | 0.21 | **0.6** |  | 231771 | 24.0mod | **3.0** | 0.21 | **0.4** |
|  |  | 55.0 |  | Pie-0.1 |  |  |  | 49.0mod |  | 0.17 |  |
|  | 230014 | 24.0 | **3.0** | 0.17mod | **0.3** |  | 231454 | 24.0mod | **1.0** | 0.21 | **0.6** |
|  |  | 49.0 |  | 0.03 |  |  |  | GMP-1.0 |  | Pie-0.1 |  |
|  | 230084 | 24.0 | **3.0** | 0.17mod | **0.3** |  | 334823 | 24.0mod | **1.0** | 0.21 | **0.6** |
|  |  | 49.0 |  | 0.03 |  |  |  | GMP-1.0 |  | Pie-0.1 |  |
|  | 332561 | 24.0 | **3.0** | 0.21 | **0.4** |  | 231604 | 24.0mod | **1.0** | 0.21 | **0.7** |
|  |  | 49.0 |  | 0.17mod |  |  |  | GMP-1.0 |  | 0.31mod |  |
|  | 332570 | 24.0 | **3.0** | 0.21 | **0.4** |  | 231383 | 10.0 | **2.0** | 0.21 | **0.6** |
|  |  | 49.0 |  | 0.17mod |  |  |  | 67.0mod |  | Pie-0.1 |  |
|  | 229930 | 55.0mod | **8.0** | 0.17mod | **0.3** |  | 334694 | 10.0 | **2.0** | 0.21 | **0.5** |
|  |  | 49.0 |  | 0.03 |  |  |  | 67.0mod |  | 0.03 |  |
|  | 230057 | 55.0mod | **8.0** | 0.17mod | **0.3** |  | 334637 | 24.0 | **1.0** | 0.21 | **0.6** |
|  |  | 49.0 |  | 0.03 |  |  |  | GMP-1.0 |  | Pie-0.1 |  |
|  | 332516 | 55.0mod | **8.0** | 0.17mod | **0.3** |  | 231615 | 24.0mod | **1.0** | 0.21 | **0.6** |
|  |  | 49.0 |  | 0.03 |  |  |  | GMP-1.0 |  | Pie-0.1 |  |
|  | 332767 | 55.0mod | **8.0** | 0.17mod | **0.3** |  | 231627 | 10.0 | **2.0** | 0.03 | **0.3** |
|  |  | 49.0 |  | 0.03 |  |  |  | 67.0mod |  | 0.17mod |  |
|  | 332387 | 55.0mod | **8.0** | 0.17mod | **0.3** |  | 334800 | 24.0mod | **3.0** | 0.21 | **0.4** |
|  |  | 49.0 |  | 0.03 |  |  |  | 49.0mod |  | 0.17mod |  |
|  | 231672 | 10.0 | **2.0** | 0.21 | **0.5** |  |  |  |  |  |  |
|  |  | 67.0mod |  | 0.03 |  |  |  |  |  |  |  |
|  | 231472 | 24.0mod | **4.0** | 0.21 | **0.5** |  |  |  |  |  |  |
|  |  | GMP-2.0 |  | 0.03 |  |  |  |  |  |  |  |

**Supplementary Table 6.** SLA-I and SLA-II genotypes and haplotypes of 209 Göttingen Minipigs (continued)

|  | **Pig ID** | **SLA-I** | **GT** | **SLA-II** | **GT** |  | **Pig ID** | **SLA-I** | **GT** | **SLA-II** | **GT** |
| --- | --- | --- | --- | --- | --- | --- | --- | --- | --- | --- | --- |
|  |  | **Lr-Hp** |  | **Lr-Hp** |  |  |  | **Lr-Hp** |  | **Lr-Hp** |  |
| **Cohort 2019** | 335829 | 24.0mod | **1.0** | 0.21 | **0.6** | **Cohort 2019** | 232510 | 24.0mod | **1.0** | 0.21 | **0.6** |
|  |  | GMP-1.0 |  | Pie-0.1 |  |  |  | GMP-1.0 |  | Pie-0.1 |  |
|  | 232336 | 10.0 | **2.0** | 0.21 | **0.5** |  | 233115 | 10.0 | **2.0** | 0.21 | **0.5** |
|  |  | 67.0mod |  | 0.03 |  |  |  | 67.0mod |  | 0.03 |  |
|  | 335840 | 10.0 | **2.0** | 0.21 | **0.5** |  | 233058 | 24.0mod | **3.0** | 0.21 | **0.4** |
|  |  | 67.0mod |  | 0.03 |  |  |  | 49.0mod |  | 0.17 |  |
|  | 232337 | 03.0mod | **5.0** | 0.03 | **0.3** |  | 337458 | 24.0mod | **3.0** | 0.21 | **0.4** |
|  |  | 49.0 |  | 0.17mod |  |  |  | 49.0mod |  | 0.17 |  |
|  | 335943 | 24.0mod | **1.0** | 0.21 | **0.6** |  | 233061 | 10.0 | **2.0** | 0.21 | **0.5** |
|  |  | GMP-1.0 |  | Pie-0.1 |  |  |  | 67.0mod |  | 0.03 |  |
|  | 232474 | 24.0mod | **4.0** | 0.21 | **0.5** |  | 337251 | 24.0mod | **1.0** | 0.21 | **0.6** |
|  |  | GMP-2.0 |  | 0.03 |  |  |  | GMP-1.0 |  | Pie-0.1 |  |
|  | 335910 | 24.0mod | **1.0** | 0.21 | **0.6** |  | 337442 | 10.0 | **7.0** | 0.21 | **0.6** |
|  |  | GMP-1.0 |  | Pie-0.1 |  |  |  | 24.0mod |  | Pie-0.1 |  |
|  | 232438 | 24.0mod | **1.0** | 0.21 | **0.6** |  | 337535 | 24.0mod | **3.0** | 0.21 | **0.4** |
|  |  | GMP-1.0 |  | Pie-0.1 |  |  |  | 49.0mod |  | 0.17 |  |
|  | 336113 | 24.0mod | **4.0** | 0.21 | **0.5** |  | 337110 | 10.0 | **2.0** | 0.21 | **0.5** |
|  |  | GMP-2.0 |  | 0.03 |  |  |  | 67.0mod |  | 0.03 |  |
|  | 232427 | 24.0mod | **1.0** | 0.21 | **0.6** |  | 337071 | 10.0 | **2.0** | 0.21 | **0.5** |
|  |  | GMP-1.0 |  | Pie-0.1 |  |  |  | 67.0mod |  | 0.03 |  |
|  | 335836 | 03.0mod | **5.0** | 0.17mod | **0.2** |  | 233163 | 03.0mod | **5.0** | 0.03 | **0.1** |
|  |  | 49.0 |  | 0.17mod |  |  |  | 49.0 |  | 0.17mod |  |
|  | 232455 | 24.0mod | **1.0** | 0.21 | **0.6** |  | 233078 | 03.0mod | **5.0** | 0.03 | **0.1** |
|  |  | GMP-1.0 |  | Pie-0.1 |  |  |  | 49.0 |  | 0.17mod |  |
|  | 335823 | 10.0 | **2.0** | 0.03 | **0.1** |  | 233292 | 10.0 | **7.0** | 0.21 | **0.5** |
|  |  | 67.0mod |  | 0.03 |  |  |  | 24.0mod |  | 0.03 |  |
|  | 232432 | 24.0mod | **3.0** | 0.21 | **0.4** |  | 337215 | 10.0 | **2.0** | 0.21 | **0.5** |
|  |  | 49.0mod |  | 0.17 |  |  |  | 67.0mod |  | 0.03 |  |
|  | 336141 | 03.0mod | **5.0** | 0.03 | **0.3** |  | 233057 | 24.0mod | **3.0** | 0.21 | **0.4** |
|  |  | 49.0 |  | 0.17mod |  |  |  | 49.0mod |  | 0.17 |  |
|  | 232499 | 10.0 | **7.0** | 0.21 | **0.5** |  | 337508 | 10.0 | **7.0** | 0.21 | **0.5** |
|  |  | 24.0 |  | 0.03 |  |  |  | 24.0mod |  | 0.03 |  |
|  | 336237 | 24.0mod | **1.0** | 0.21 | **0.6** |  | 233184 | 24.0mod | **1.0** | 0.21 | **0.6** |
|  |  | GMP-1.0 |  | Pie-0.1 |  |  |  | GMP-1.0 |  | Pie-0.1 |  |

**Supplementary Table 6.** SLA-I and SLA-II genotypes and haplotypes of 209 Göttingen Minipigs (continued)

|  | **Pig ID** | **SLA-I** | **GT** | **SLA-II** | **GT** |  | **Pig ID** | **SLA-I** | **GT** | **SLA-II** | **GT** |
| --- | --- | --- | --- | --- | --- | --- | --- | --- | --- | --- | --- |
|  |  | **Lr-Hp** |  | **Lr-Hp** |  |  |  | **Lr-Hp** |  | **Lr-Hp** |  |
| **Cohort 2019** | 337540 | 24.0mod | **3.0** | 0.21 | **0.4** | **Cohort 2019** | 234131 | 24.0 | **3.0** | 0.03 | **0.3** |
|  |  | 49.0mod |  | 0.17 |  |  |  | 49.0 |  | 0.17mod |  |
|  | 337476 | 03.0mod | **5.0** | 0.03 | **0.3** |  | 234230 | 24.0mod | **1.0** | 0.21 | **0.7** |
|  |  | 49.0 |  | 0.17mod |  |  |  | GMP-1.0 |  | 0.31mod |  |
|  | 234364 | 24.0 | **17.0** | 0.21 | **0.6** |  | 234164 | 03.0mod | **11.0** | 0.03 | **0.1** |
|  |  | 24.0 |  | Pie-0.1 |  |  |  | 24.0 |  | 0.03 |  |
|  | 234163 | 03.0mod | **11.0** | 0.03 | **0.1** |  | 333304 | 10.0 | **7.0** | 0.21 | **0.5** |
|  |  | 24.0 |  | 0.03 |  |  |  | 24.0mod |  | 0.03 |  |
|  | 339148 | 03.0mod | **11.0** | 0.03 | **0.1** |  | 333504 | 24.0 | **17.0** | 0.21 | **0.6** |
|  |  | 24.0 |  | 0.03 |  |  |  | 24.0 |  | Pie-0.1 |  |
|  | 338853 | 24.0 | **3.0** | 0.21 | **0.6** |  | 335055 | 10.0 | **7.0** | 0.21 | **0.5** |
|  |  | 49.0 |  | Pie-0.1 |  |  |  | 24.0mod |  | 0.03 |  |
|  | 338759 | 3.0 | **11.0** | 0.21 | **0.5** |  | 340259 | 24.0 | **17.0** | 0.21 | **0.6** |
|  |  | 24.0mod |  | 0.03 |  |  |  | 24.0 |  | Pie-0.1 |  |
|  | 234140 | 10.0 | **2.0** | 0.21 | **0.5** |  | 340246 | 24.0 | **3.0** | 0.21 | **0.4** |
|  |  | 67.0mod |  | 0.03 |  |  |  | 49.0 |  | 0.17 |  |
|  | 234361 | 10.0 | **2.0** | 0.21 | **0.5** |  | 340255 | 03.0mod | **5.0** | 0.03 | **0.3** |
|  |  | 67.0mod |  | 0.03 |  |  |  | 49.0 |  | 0.17mod |  |
|  | 338800 | 24.0mod | **3.0** | 0.21 | **0.4** |  | 340221 | 24.0 | **17.0** | 0.21 | **0.6** |
|  |  | 49.0mod |  | 0.17 |  |  |  | 24.0 |  | Pie-0.1 |  |
|  | 234375 | 10.0 | **2.0** | 0.21 | **0.6** |  | 340219 | 24.0 | **17.0** | 0.21 | **0.6** |
|  |  | 67.0mod |  | Pie-0.1 |  |  |  | 24.0 |  | Pie-0.1 |  |
|  | 338825 | 24.0 | **17.0** | 0.21 | **0.6** |  | 340257 | 10.0 | **7.0** | 0.21 | **0.5** |
|  |  | 24.0 |  | Pie-0.1 |  |  |  | 24.0mod |  | 0.03 |  |
|  | 338912 | 24.0mod | **1.0** | 0.21 | **0.6** |  | 340253 | 03.0mod | **5.0** | 0.21 | **0.4** |
|  |  | GMP-1.0 |  | Pie-0.1 |  |  |  | 49.0 |  | 0.17 |  |
|  | 338845 | 10.0 | **2.0** | 0.21 | **0.5** |  | 340247 | 24.0 | **3.0** | 0.21 | **0.5** |
|  |  | 67.0mod |  | 0.03 |  |  |  | 49.0 |  | 0.03 |  |
|  | 234121 | 24.0mod | **1.0** | 0.21 | **0.7** |  | 340226 | 24.0 | **17.0** | 0.21 | **0.6** |
|  |  | GMP-1.0 |  | 0.31mod |  |  |  | 24.0 |  | Pie-0.1 |  |
|  | 234089 | 24.0mod | **3.0** | 0.21 | **0.4** |  | 340222 | 24.0 | **17.0** | 0.21 | **0.6** |
|  |  | 49.0mod |  | 0.17 |  |  |  | 24.0 |  | Pie-0.1 |  |
|  | 234202 | 03.0mod | **11.0** | 0.03 | **0.1** |  |  |  |  |  |  |
|  |  | 24.0 |  | 0.03 |  |  |  |  |  |  |  |

**Supplementary Table 6.** SLA-I and SLA-II genotypes and haplotypes of 209 Göttingen Minipigs (continued)

|  | **Pig ID** | **SLA-I** | **GT** | **SLA-II** | **GT** |  | **Pig ID** | **SLA-I** | **GT** | **SLA-II** | **GT** |
| --- | --- | --- | --- | --- | --- | --- | --- | --- | --- | --- | --- |
|  |  | **Lr-Hp** |  | **Lr-Hp** |  |  |  | **Lr-Hp** |  | **Lr-Hp** |  |
| **Cohort 2021** | 333637 | 17.0 | **24.0** | 0.17 | **0.4** | **Cohort 2021** | 333645 | GMP-3.0 | **20.0** | 0.21 | **0.6** |
|  |  | 49.0 |  | 0.21 |  |  |  | GMP-1.0 |  | Pie-0.1 |  |
|  | 333711 | GMP-3.0 | **18.0** | 0.03 | **0.5** |  | 333674 | 3.0 | **12.0** | 0.03 | **0.5** |
|  |  | GMP-3.0 |  | 0.21 |  |  |  | GMP-3.0 |  | 0.21 |  |
|  | 333608 | 17.0 | **26.0** | 0.03 | **0.3** |  | 333712 | GMP-3.0 | **21.0** | 0.03 | **0.5** |
|  |  | 44.0mod |  | 0.17mod |  |  |  | 67.0mod |  | 0.21 |  |
|  | 333641 | GMP-3.0 | **22.0** | 0.17 | **0.4** |  | 333722 | 10.0 | **16.0** | 0.03 | **0.3** |
|  |  | 49.0 |  | 0.21 |  |  |  | GMP-3.0 |  | 0.17mod |  |
|  | 333713 | 10.0 | **16.0** | 0.03 | **0.5** |  | 333749 | GMP-3.0 | **18.0** | 0.21 | **0.6** |
|  |  | GMP-3.0 |  | 0.21 |  |  |  | GMP-3.0 |  | Pie-0.1 |  |
|  | 333692 | GMP-3.0 | **18.0** | 0.03 | **0.1** |  | 339280 | 3.0 | **5.0** | 0.03 | **0.1** |
|  |  | GMP-3.0 |  | 0.03 |  |  |  | 49.0 |  | 0.03 |  |
|  | 333669 | GMP-3.0 | **20.0** | 0.21 | **0.6** |  | 339145 | GMP-3.0 | **20.0** | 0.21 | **0.6** |
|  |  | GMP-1.0 |  | Pie-0.1 |  |  |  | GMP-1.0 |  | Pie-0.1 |  |
|  | 333745 | GMP-3.0 | **18.0** | 0.21 | **0.6** |  | 4163 | GMP-3.0 | **21.0** | 0.03 | **0.5** |
|  |  | GMP-3.0 |  | Pie-0.1 |  |  |  | 67.0mod |  | 0.21 |  |
|  | 333686 | GMP-3.0 | **18.0** | 0.03 | **0.1** |  | 4174 | GMP-3.0 | **18.0** | 0.03 | **0.5** |
|  |  | GMP-3.0 |  | 0.03 |  |  |  | GMP-3.0 |  | 0.21 |  |
|  | 333615 | GMP-3.0 | **22.0** | 0.21 | **0.6** |  | 4170 | GMP-3.0 | **21.0** | 0.03 | **0.1** |
|  |  | 49.0mod |  | Pie-0.1 |  |  |  | 67.0mod |  | 0.03 |  |
|  | 228504 | 10.0 | **16.0** | 0.03 | **0.5** |  | 4184 | 3.0 | **10.0** | 0.03 | **0.1** |
|  |  | GMP-3.0 |  | 0.21 |  |  |  | 3.0 |  | 0.03 |  |
|  | 329952 | GMP-3.0 | **22.0** | 0.17 | **0.4** |  | 4180 | 03.0mod | **5.0** | 0.03 | **0.3** |
|  |  | 49.0mod |  | 0.21 |  |  |  | 49.0mod |  | 0.17mod |  |
|  | 330041 | 3.0 | **9.0** | 0.03 | **0.3** |  | 4191 | 4.0 | **25.0** | 0.21 | **0.6** |
|  |  | 17.0 |  | 0.17mod |  |  |  | GMP-3.0 |  | Pie-0.1 |  |
|  | 234341 | GMP-3.0 | **20.0** | 0.21 | **0.6** |  | 4169 | GMP-3.0 | **21.0** | 0.03 | **0.5** |
|  |  | GMP-1.0 |  | Pie-0.1 |  |  |  | 67.0mod |  | 0.21 |  |
|  | 231975 | 17.0 | **26.0** | 0.03 | **0.3** |  | 4177 | GMP-3.0 | **18.0** | 0.03 | **0.5** |
|  |  | 44.0mod |  | 0.17mod |  |  |  | GMP-3.0 |  | 0.21 |  |
|  | 232107 | 3.0 | **12.0** | 0.03 | **0.3** |  | 4182 | GMP-3.0 | **22.0** | 0.21 | **0.4** |
|  |  | GMP-3.0 |  | 0.17mod |  |  |  | 49.0mod |  | 0.17mod |  |
|  | 232004 | 03.0mod | **12.0** | 0.3 | **0.5** |  | 4185 | 3.0 | **10.0** | 0.03 | **0.1** |
|  |  | GMP-3.0 |  | 0.21 |  |  |  | 3.0 |  | 0.03 |  |

**Supplementary Table 6.** SLA-I and SLA-II genotypes and haplotypes of 209 Göttingen Minipigs (continued)

|  | **Pig ID** | **SLA-I** | **GT** | **SLA-II** | **GT** |  | **Pig ID** | **SLA-I** | **GT** | **SLA-II** | **GT** |
| --- | --- | --- | --- | --- | --- | --- | --- | --- | --- | --- | --- |
|  |  | **Lr-Hp** |  | **Lr-Hp** |  |  |  | **Lr-Hp** |  | **Lr-Hp** |  |
| **Cohort 2023** | 500299 | GMP-3.0 | **20.0** | 0.03 | **0.5** | **Cohort 2023** | 244362 | GMP-3.0 | **20.0** | 0.21 | **0.6** |
|  |  | GMP-1.0 |  | 0.21 |  |  |  | GMP-1.0 |  | Pie-0.1 |  |
|  | 500270 | GMP-3.0 | **22.0** | 0.17 | **0.4** |  | 244484 | GMP-3.0 | **20.0** | 0.03 | **0.5** |
|  |  | 49.0mod |  | 0.21 |  |  |  | GMP-1.0 |  | 0.21 |  |
|  | 500370 | GMP-3.0 | **21.0** | 0.21 | **0.6** |  | 244319 | GMP-3.0 | **20.0** | 0.03 | **0.5** |
|  |  | 67.0mod |  | Pie-0.1 |  |  |  | GMP-1.0 |  | 0.21 |  |
|  | 500378 | GMP-3.0 | **18.0** | 0.03 | **0.1** |  | 244473 | GMP-3.0 | **22.0** | 0.17mod | **0.4** |
|  |  | GMP-3.0 |  | 0.03 |  |  |  | 49.0mod |  | 0.21 |  |
|  | 500363 | GMP-3.0 | **22.0** | 0.03 | **0.3** |  | 243595 | GMP-3.0 | **20.0** | 0.03 | **0.5** |
|  |  | 49.0 |  | 0.17mod |  |  |  | GMP-1.0 |  | 0.21 |  |
|  | 244131 | GMP-3.0 | **22.0** | 0.17 | **0.4** |  | 243405 | GMP-3.0 | **18.0** | 0.21 | **0.6** |
|  |  | 49.0 |  | 0.21 |  |  |  | GMP-3.0 |  | Pie-0.1 |  |
|  | 244980 | GMP-3.0 | **20.0** | 0.03 | **0.1** |  | 243809 | 10.0 | **16.0** | 0.03 | **0.5** |
|  |  | GMP-1.0 |  | 0.03 |  |  |  | GMP-3.0 |  | 0.21 |  |
|  | 244251 | GMP-3.0 | **21.0** | 0.21 | **0.7** |  | 244178 | 10.0 | **16.0** | 0.03 | **0.5** |
|  |  | 67.0mod |  | 0.31mod |  |  |  | GMP-3.0 |  | 0.21 |  |
|  | 243893 | GMP-3.0 | **20.0** | 0.21 | **0.6** |  | 243829 | GMP-3.0 | **19.0** | 0.03 | **0.5** |
|  |  | GMP-1.0 |  | Pie-0.1 |  |  |  | GMP-2.0 |  | 0.21 |  |
|  | 244961 | GMP-3.0 | **18.0** | 0.03 | **0.5** |  | 244336 | GMP-3.0 | **19.0** | 0.21 | **0.6** |
|  |  | GMP-3.0 |  | 0.21 |  |  |  | GMP-2.0 |  | Pie-0.1 |  |
|  | 243855 | 10.0 | **16.0** | 0.03 | **0.5** |  | 351486 | GMP-3.0 | **20.0** | 0.03 | **0.5** |
|  |  | GMP-3.0 |  | 0.21 |  |  |  | GMP-1.0 |  | 0.21 |  |
|  | 244631 | 49.0 | **23.0** | 0.17mod | **0.2** |  | 351771 | GMP-3.0 | **16.0** | 0.21 | **0.6** |
|  |  | 49.0 |  | 0.17mod |  |  |  | 10.0 |  | Pie-0.1 |  |
|  | 243911 | 10.0 | **15.0** | 0.03 | **0.3** |  | 352290 | GMP-3.0 | **16.0** | 0.21 | **0.6** |
|  |  | 49.0 |  | 0.17mod |  |  |  | 10.0 |  | Pie-0.1 |  |
|  | 243641 | GMP-3.0 | **22.0** | 0.17mod | **0.4** |  | 351513 | GMP-3.0 | **16.0** | 0.21 | **0.6** |
|  |  | 49.0mod |  | 0.21 |  |  |  | 10.0 |  | Pie-0.1 |  |
|  | 244058 | GMP-3.0 | **22.0** | 0.17mod | **0.4** |  | 352473 | GMP-3.0 | **20.0** | 0.03 | **0.5** |
|  |  | 49.0mod |  | 0.21 |  |  |  | GMP-1.0 |  | 0.21 |  |
|  | 243973 | GMP-3.0 | **20.0** | 0.03 | **0.5** |  | 352498 | GMP-3.0 | **20.0** | 0.03 | **0.5** |
|  |  | GMP-1.0 |  | 0.21 |  |  |  | GMP-1.0 |  | 0.21 |  |

**Supplementary Table 6.** SLA-I and SLA-II genotypes and haplotypes of 209 Göttingen Minipigs (continued)

|  | **Pig ID** | **SLA-I** | **GT** | **SLA-II** | **GT** |
| --- | --- | --- | --- | --- | --- |
|  |  | **Lr-Hp** |  | **Lr-Hp** |  |
| **Cohort 2023** | 351120 | GMP-3.0 | **20.0** | 0.21 | **0.6** |
|  |  | GMP-1.0 |  | Pie-0.1mod |  |
|  | 350675 | GMP-3.0 | **21.0** | 0.03 | **0.5** |
|  |  | 67.0mod |  | 0.21 |  |
|  | 349928 | GMP-3.0 | **20.0** | 0.21 | **0.6** |
|  |  | GMP-1.0 |  | Pie-0.1mod |  |
|  | 351607 | GMP-3.0 | **20.0** | 0.03 | **0.3** |
|  |  | GMP-1.0 |  | 0.17mod |  |
|  | 353100 | YDLR-2.0 | **14.0** | 0.21 | **0.7** |
|  |  | 49.0mod |  | 0.31mod |  |
|  | 351146 | GMP-3.0 | **18.0** | 0.03 | **0.5** |
|  |  | GMP-3.0 |  | 0.21 |  |
|  | 351483 | 10.0 | **15.0** | 0.17mod | **0.4** |
|  |  | 49.0mod |  | 0.21 |  |
|  | 351690 | GMP-3.0 | **20.0** | 0.03 | **0.5** |
|  |  | GMP-1.0 |  | 0.21 |  |
|  | 352478 | 5.0 | **13.0** | 0.21 | **0.6** |
|  |  | GMP-3.0 |  | Pie-0.1 |  |
|  | 352149 | GMP-3.0 | **20.0** | 0.03 | **0.5** |
|  |  | GMP-1.0 |  | 0.21 |  |
|  | 353087 | GMP-3.0 | **16.0** | 0.21 | **0.6** |
|  |  | 10.0 |  | Pie-0.1 |  |
|  | 351853 | GMP-3.0 | **16.0** | 0.21 | **0.6** |
|  |  | 10.0 |  | Pie-0.1 |  |
|  | 352069 | GMP-3.0 | **16.0** | 0.21 | **0.6** |
|  |  | 10.0 |  | Pie-0.1 |  |

ELLG = Ellegaard; SLA-Swine Leucocyte Antigen; Lr-Hp = Low resolution Haplotype; GT = Genotype; mod = modified; GMP = Göttingen Minipig; Pie = Pietrain; YDLR = Yorkshire/Duroc/Landrace 3-way crossbreed.

**Supplementary Table 7.** Shared haplotypes of Göttingen Minipigs and European Farmed pigs

|  |  | **Göttingen Minipigs** | **Farmed pigs** |
| --- | --- | --- | --- |
|  | **Haplotype** | **Frq(%)** | **Frq(%)** |
| **SLA class I** | Lr-03.0 | 5.98 | 0.00 |
|  | Lr-04.0 | 0.24 | 11.02 |
|  | Lr-05.0 | 0.24 | 0.91 |
|  | Lr-10.0 | 10.77 | 0.00 |
|  | Lr-17.0 | 0.96 | 0.00 |
|  | Lr-24.0 | 23.44 | 5.02 |
|  | Lr-44.0 | 0.48 | 0.00 |
|  | Lr-49.0 | 14.59 | 1.55 |
|  | Lr-55.0 | 5.50 | 1.83 |
|  | Lr-67.0 | 6.94 | 0.29 |
|  | Lr-GMP-1.0 | 10.29 | 0.00 |
|  | Lr-GMP-2.0 | 1.67 | 0.00 |
|  | Lr-GMP-3.0 | 18.66 | 0.00 |
|  | Lr-YDLR-2.0 | 0.24 | 0.00 |
| **SLA class II** | Lr-0.03 | 30.38 | 0.00 |
|  | Lr-0.17 | 16.27 | 0.00 |
|  | Lr-0.21 | 38.28 | 6.01 |
|  | Lr-0.31 | 1.20 | 0.00 |
|  | Lr-Pie-0.1 | 13.88 | 0.15 |

SLA = Swine Leucocyte Antigen; Lr = Low resolution Haplotype; GMP = Göttingen Minipig; YDLR = Yorkshire/Duroc/Landrace 3-way crossbreed; Pie = Pietrain; Freq = frequency (in %). Reference: Hammer, S.E., Duckova, T., Groiss, S., Stadler, M., Jensen-Waern, M., Golde, W.T., Gimsa, U., Saalmüller, A. (2021). Comparative analysis of swine leukocyte antigen gene diversity in European farmed pigs. Animal Genetics. 52(4): 523-531. https://doi.org/10.1111/age.13090.

**Supplementary Table 8.** Shared allele groups of Göttingen Minipigs and European Farmed pigs

|  |  |  | **Göttingen Minipigs** | **Farmed pigs** |
| --- | --- | --- | --- | --- |
|  |  | **Allele group** | **Frq (%)** | **Frq (%)** |
| **SLA class I** | **SLA-1** | 04XX | 0.48 | 12.26 |
|  |  | 05XX | 10.77 | 0.00 |
|  |  | 06XX | 0.48 | 0.00 |
|  |  | 08XX | 15.55 | 16.12 |
|  |  | 05XX/15XX | 23.68 | 0.00 |
|  |  | 15XX | 14.83 | 3.67 |
|  |  | 16:02(16:03) | 18.90 | 0.39 |
|  |  | Blank or Null | 15.31 | 15.06 |
|  | **SLA-3** | 03XX(03:04)/08XX | 25.60 | 5.31 |
|  |  | 04XX | 29.43 | 39.00 |
|  |  | 05XX/08XX | 32.54 | 22.49 |
|  |  | 08XX | 12.44 | 0.10 |
|  | **SLA-2** | 01XX/03XX | 18.90 | 7.82 |
|  |  | 03XX | 17.22 | 1.64 |
|  |  | 04XX | 0.24 | 14.77 |
|  |  | 06XX | 30.62 | 10.14 |
|  |  | 08XX | 0.24 | 0.48 |
|  |  | 11:04 | 5.50 | 4.05 |
|  |  | 03XX/17:01 | 18.66 | 0.00 |
|  |  | Blank | 8.61 | 1.35 |
| **SLA class II** | **DRB1** | 01XX | 52.15 | 11.45 |
|  |  | 03XX(03:02) | 31.58 | 0.00 |
|  |  | 08XX | 16.27 | 1.13 |
|  | **DQB1** | 03XX(03:01) | 30.38 | 2.26 |
|  |  | 05XX | 69.62 | 7.42 |
|  | **DQA** | 01XX | 30.86 | 33.87 |
|  |  | 04XX(+05XX) | 51.44 | 11.45 |
|  |  | Blank or Null | 17.70 | 0.16 |

SLA = Swine Leucocyte Antigen; Blank = Indicating alleles that cannot be identified with the study primer sets.; Freq = frequency (in %). Reference: Hammer, S.E., Duckova, T., Groiss, S., Stadler, M., Jensen-Waern, M., Golde, W.T., Gimsa, U., Saalmüller, A. (2021). Comparative analysis of swine leukocyte antigen gene diversity in European farmed pigs. Animal Genetics. 52(4): 523-531. https://doi.org/10.1111/age.13090.
